# Carbon dots and metal–organic frameworks based nanohybrids for improved biosensing and biomedical applications

**DOI:** 10.1039/d5na00950b

**Published:** 2026-02-06

**Authors:** Tania P. Brito, Leonel Llanos, Dinesh P. Singh

**Affiliations:** a Physics Department, Faculty of Science, University of Santiago of Chile (USACH) Avenida Victor Jara 3493, Estación Central Santiago 9170124 Chile tania.brito@usach.cl singh.dinesh@usach.cl; b Faculty of Chemical and Pharmaceutical Sciences, University of Chile Olivos 1007 8380544 Santiago Chile leonel.llanos@usach.cl

## Abstract

Composites of carbon dots (CDs) and metal–organic frameworks (CDs@MOFs) have emerged as a novel class of hybrid nanomaterials that integrate the high porosity and structural versatility of MOFs with the outstanding optical properties and biocompatibility of CDs. Although relatively new, these materials have attracted growing interest as multifunctional platforms for applications in biosensing of bioactive molecules, drugs, and biomarkers, as well as in cancer diagnosis and treatment and antibacterial activity, thereby expanding their potential in biomedicine. The promising performance demonstrated in these areas underscores the need to review and analyze recent advances, along with the benefits and challenges associated with this new class of materials. This review aims to provide a summary of the progress made in the synthesis of CDs@MOFs, including the methodologies and precursors employed, and highlights how the synthesis approach directly influences material properties and guides the selection of the appropriate CDs@MOFs hybrid material based on the desired application. Subsequently, the applications of CDs@MOFs in biosensors for the detection of bioactive molecules, drugs, and biomarkers are discussed, emphasizing advances in detection mechanisms, analytical performance, and stability. Their emerging applications in cancer diagnosis and treatment, antibacterial activity, and wound therapy are also reviewed. Finally, current challenges are discussed, including the control of CD distribution within MOF structures, stability in physiological media, and the need for sustainable and cost-effective synthesis methods, along with an overview of future research and development opportunities.

## Abstract 2

Nanocomposites of carbon dots and metal–organic frameworks (CDs@MOFs) have emerged as a novel class of materials that integrate the high porosity and structural versatility of MOFs with the outstanding optical properties and biocompatibility of CDs.

## Graphical abstract

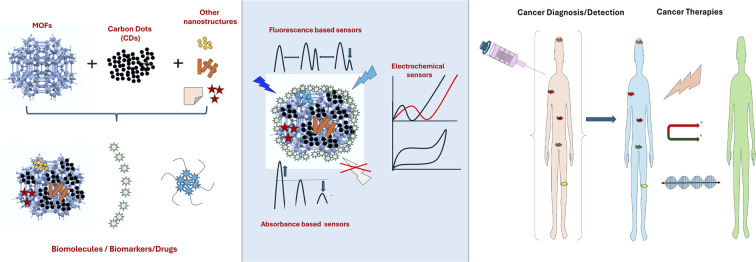

## 1. Introduction

Since the consolidation of nanomaterials research in the 1990s, incredible advances have been made around the world, with a great variety of structures showing diverse and tunable properties (electrical, mechanical, optical, and chemical).^1^ Among the most studied are carbon-derived nanomaterials (graphene, carbon nanotubes, fullerenes, carbon dots, and nanodiamonds), and metal-oxide, -phosphide, and -sulfide nanoparticles.^2,3^ Furthermore, nanoporous materials such as metal–organic frameworks (MOFs), covalent organic frameworks (COFs), and zeolites have been extensively studied. The rapid evolution of each class of material intertwines with the development of composites among them, allowing synergistic interactions that can be designed to enhance their properties. In this way, nanomaterials have been further exploited in biological applications, ranging from biomaterial development, disease treatment, and theranostics to biosensing, with state-of-the-art performance at present.^4,5^

The development of MOF-based materials comprises a constantly growing field spanning a wide range of applications,^6^ such as catalysis,^7^ photonics,^8^ sensing,^9,10^ energy storage,^11^ drug delivery,^12^ magnetic materials,^13^ and environmentally friendly technologies.^14^ This is mainly due to the high tunability of their metal centers (nodes) and ligands (linkers), allowing the preparation of highly stable extended materials with carefully designed chemical or optical properties. One of the unique features of MOFs is their chemically controlled pore size and interactions inside the surface and cavity,^15^ enabling them to host molecules, either to improve MOF properties or to accumulate analytes.

Thus, electrochemical^16^ and optical^17^ MOF-based sensors are promising devices that cover a wide range of analytes and detection ranges, including biologically relevant targets such as biomolecules, drugs, and biomarkers.^18,19^ Regarding their optical properties, photoluminescence (PL) is commonly used as a probe signal sensitive to interactions between MOFs and other molecules, which can originate from the organic linkers or involve the metal centers, and is often quenched by the presence of the analyte.^20^ In addition, these materials have been explored in other biological applications, such as cancer treatment and diagnosis, where MOFs can act as efficient drug delivery systems, contrast agents for imaging, and platforms for photodynamic or photothermal therapy.^21^ They have also demonstrated significant antibacterial activity, either by the sustained release of biocidal metal ions, such as Zn^2+^, Cu^2+^, or Ag^+^, or through the production of reactive oxygen species (ROS) under light irradiation, making them suitable for infection control and wound-healing applications.^22,23^

Although MOFs can extend to one, two, or three dimensions, carbon dots (CDs) have emerged as a fascinating family of zero-dimensional nanomaterials derived from graphitic nanosheets, with particle sizes of around 10 nm and highly tunable sizes, morphologies, and optical properties.^24,25^ Unlike nonluminescent graphene, CDs are typically highly emissive under light excitation,^26^ which is attributed to quantum confinement effects. In this regard, doping with heteroatoms/metals or surface functionalization with different chemical groups shows improved optical properties such as different PL colors from blue to the NIR region, including the biological window.^27^ In this sense, CDs emitting in the near-infrared (NIR) region are highly relevant for bioimaging, biosensing, and drug delivery applications.^28,29^ This property is especially valuable in *in vivo* studies because body tissues exhibit greater transparency in this spectral window. Unlike UV radiation, which is harmful, and visible light, which is easily absorbed by biological tissues, NIR radiation offers deeper and safer penetration.^30,31^

CDs exhibit enriched photophysics due to the interplay between core/surface carbon-related states and their interaction with dopants or chemical groups, which contribute new holes/electrons affecting luminescence.^32^ Moreover, chemical modifications lead to fine-tuning of their interactions with other molecules, which can accept excited-state energy or electrons for different applications.

Hence, CD-derived materials have applications in optoelectronics^33,34^ and sensing^35^ while also standing out owing to their low toxicity, high biocompatibility, tunable optical properties, and ease of functionalization, which make them especially attractive for biomedical uses.^36^ Beyond these areas, CDs are valued for their reduced production costs, simple synthesis, excellent water solubility, and remarkable photochemical stability against photobleaching.^37–39^ These characteristics underpin their suitability for diverse biomedical applications, ranging from the selective detection of biomolecules and biomarkers in sensitive bioassays to their intrinsic peroxidase-like activity, which has been exploited in enzyme-mimicking biosensors and catalytic therapeutic platforms.^40^ Additionally, CDs have been utilized as contrast agents for bioimaging^41^ and as nanocarriers for controlled drug delivery,^42^ thereby contributing to advancements in cancer diagnosis and therapy.^43,44^ Furthermore, their ability to generate ROS under light irradiation, combined with their versatile surface chemistry, has been leveraged in antibacterial strategies, underscoring their multifunctional potential in biomedicine.^45^

Because MOFs and CDs have remarkable features that can be harnessed in several applications, it is natural to aim toward the development of CDs@MOFs composites seeking synergistic effects.^46^ The first study concerning the hybridization of both materials was reported in 2013, where 5 nm graphene quantum dots (GQDs) were encapsulated inside a zeolitic imidazolate framework (ZIF-8) through an *in situ* procedure.^47^ The reported material showed a bathochromic shift (32 nm) in the CD luminescence, improved stability, and increased capacity for water adsorption compared to the pristine MOF. Later, in 2014, the same MOF structure was applied to host an amine-capped CD (4–8 nm) exhibiting high CD-based PL, which can be quenched by Cu^2+^ ions accumulated inside the MOF, reaching a very low detection limit (80 pM) and a wide response range (2–1000 nM).^48^ After these publications, several CDs@MOFs composites were developed through different synthesis methodologies, highlighting significant improvements in their chemical and optical properties compared to individual MOFs or CDs.^49^ Currently, various synthesis strategies are employed for obtaining CDs@MOFs, with the most widely used approaches, particularly in the biomedical field, being bottle-around-ship, physical mixing, and one-pot synthesis.^50^ Each of these approaches presents specific advantages and limitations; thus, the selection of the method directly depends on the intended application of the hybrid material.

Although the first report on CDs@MOFs was published in 2013, most biological applications using this family of materials have been reported over the last five years. Thus, the growing interest and performance of CDs@MOFs in biosensing and biomedical research stimulated the present review, where the connection between material structure and its corresponding application is considered. Different synthetic methodologies for these composites are described. Emphasis has been placed on biosensing studies owing to the high number of articles employing CDs@MOFs as sensors for biomolecules, drugs, and biomarkers. This section is separated by different analytes, for which the reported analytical methodology and detection parameters are compiled. Moreover, their applications in the areas of cancer diagnosis, cancer treatment, and antibacterial therapy are discussed, describing the different working mechanisms used and the function of both the MOF and the different CDs used. Hence, this review focuses on studies reporting the biomedical applications of CDs@MOFs-based hybrid materials, analysing their advantages and limitations, as well as their potential contribution to future applications.

## 2. Synthesis and modification strategies for CDs@MOFs hybrid materials

**Fig. 1:**
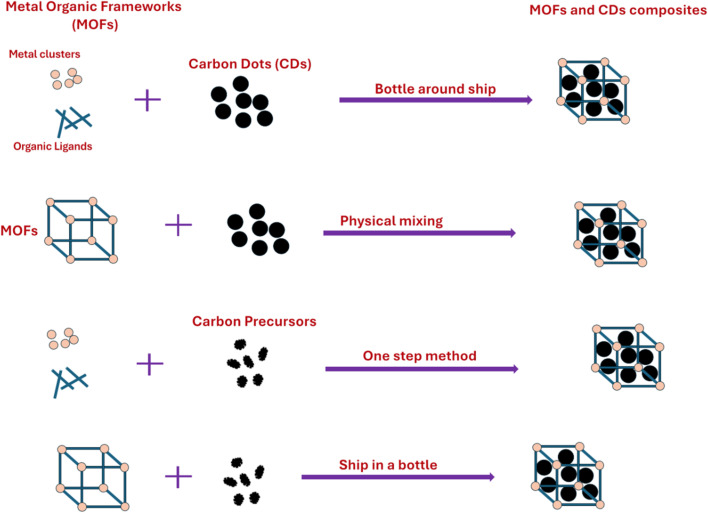
Schematic of different strategies for the synthesis of the CDs@MOFs hybrid material.

CDs and MOFs are two families of materials with an enormous number of possible variations in their chemical composition, structure, morphology, and particle size, leading to a wide chemical space for the exploitation of the properties of CDs@MOFs. However, the development of CDs@MOFs relies on different synthetic approaches, which aim to ensure the interaction between CDs and MOFs and the homogeneity of the material. In this way, the two- and one-step methods have emerged as the most common strategies to synthesize hybrid materials with optical/electrochemical properties for bioanalytical applications. Fig. 1 shows the schematic of the different strategies employed for the synthesis of the CDs@MOFs hybrid material, which are further described in detail.

### 2.1. Two-step method

The two-step method facilitates the production of the CDs@MOFs composite through the “bottle-around-ship” method, where the previously synthesized CDs are integrated into the MOF synthesis process using their respective precursors.^51^ Conversely, the “physical mixing method” involves non-covalent interactions between the components, combining the previously synthesized CDs and MOFs.^52^

#### 2.1.1. Bottle-around-ship

In this approach, as shown in Fig. 1, CDs, which have already been synthesized, are added to the solution of MOF precursors, allowing the formation of the MOF under certain conditions. This facilitates the adsorption of CDs into the cavities of the porous MOF.^53^

This method is one of the most commonly used to obtain CDs@MOFs composites, which are used in various applications such as biomolecule sensors,^54–56^ drugs,^57,58^ antibiotics,^59,60^ biomarkers,^61,62^ cancer treatments,^63^ and as antibacterial agents.^64^ However, it is necessary to apply optimal synthesis conditions to avoid quenching of the CD emission by the MOF, and it requires a rational and controlled design to optimize the final properties of the composite.

In this context, Kelin Hu *et al.*^65^ designed a non-enzymatic butyrylcholinesterase (BChE) sensor composed of CDs@ZIF-8. This material was synthesized using the bottle-around-ship method. In this approach, 2-methylimidazole (2-MeIm), Zn(CH_3_COOH)_2_, and CDs were mixed in a DMF solution and stirred for 5 minutes. The CDs were previously synthesized using a hydrothermal process at 200 °C for 5 h, employing citric acid and *o*-phenylenediamine (OPD) as precursors. This synthesis method resulted in a decrease in the fluorescence emission of the CDs encapsulated in the ZIF-8 structure. However, the competitive interaction between BChE and the metallic nodes of ZIF-8 promotes the release of the CDs, allowing for BChE quantification through fluorescent signal recovery. XPS and FTIR analyses confirmed that the chemical composition and valence state of ZIF-8 were preserved after the addition of CDs. Furthermore, the XRD diffraction patterns showed similar diffraction peaks for the CDs@ZIF-8 material, indicating that the crystal structure of ZIF-8 remained intact after the addition of the CDs. These results indicated the effective incorporation of the CDs into the ZIF-8 structure, which also maintained its composition and morphology after synthesis.

Another interesting study that utilized this synthetic method was reported by Liu J.-Y. *et al.*,^66^ who incorporated chiral CDs (CCDs), which are particularly attractive for the detection of chiral compounds due to their intrinsic chirality. In their study, CCDs enabled the development of an activatable fluorescent probe for the selective detection of d and l folic acid (FA) and the antibiotic nitrofurazone (NFZ) in real samples. The CCDs were synthesized *via* the hydrothermal method using l-cysteine and citric acid as precursors at 160 °C for 6 hours. The CCDs were then incorporated into the ZIF-8 structure by mixing them with Zn(NO_3_)_2_ and 2-MeIm in ethanol, and the mixture was stirred for 19 hours at room temperature. FTIR analysis revealed that the polar groups (–OH and –COOH) of the CCDs can form hydrogen bonds with the imidazole nitrogen atoms in ZIF-8 and also exhibit weak interactions with the MOF structure. TEM images showed a high density of CCDs encapsulated in ZIF-8, with crystal planes of 0.41 nm, consistent with an average particle size of 3.3 nm. Fluorescence characterization confirmed the successful encapsulation of the CCDs, showing characteristic emission at 445 nm under excitation at 395 nm, similar to free CCDs. The structure and luminescent properties of the CCDs@ZIF-8 material remained stable after 24 hours of immersion in water and ethanol.

Different synthetic approaches for CDs@MOFs hybrid materials are summarized in Table 1.

**Table 1:** Synthesis of CDs@MOFs hybrid materials and their applications

Material	Synthesis	Fluorescence emission wavelength	Particle size of CDs (nm)	Particle size of CDs@MOFs (nm)	Application	Ref.
CDs@ZIF-8	Bottle-around-ship	436 nm (CD)	—	250	Sensing of butyrylcholinesterase (BchE)	65
CCDs@ZIF-8	Bottle-around-ship	445 nm (CD)	3.3	—	Sensing of folic acid (FA) and nitrofurazone (NFZ)	66
		(*λ*_exc_ = 395 nm)				
S-CDs, N-CDs@AgMOF	Bottle-around-ship	—	—	100–400	Antibacterial activity	64
CDs@Eu-MOF	Bottle-around-ship	436 nm (CD)	2	—	Sensing of doxycycline (DOX)	67
		616 nm (MOF-Eu)				
		(*λ*_exc_ = 298 nm)				
PEI@CDs@Ni-MOF	Bottle-around-ship	—	16.3–44 nm	—	Cancer diagnosis	68
CDs@ZIF-8	Bottle-around-ship	448 nm (CD)	3 nm	244	Antibacterial activity	69
		(*λ*_exc_ = 398 nm)				
GOx/CDs@MOF NFs	Bottle-around-ship	435 nm (CD)	4.8 nm	—	Antibacterial activity and diabetic wound healing	70
		(*λ*_exc_ = 365 nm)				
y-CD/Cr-MOF@MIPs	Physical mixing	450 nm (Cr-MOF) and 560 nm (y-CD)	—	25–788	Sensing of uric acid (UA)	71
		(*λ*_exc_ = 390 nm)			Ascorbic acid (AA)	
g-CDs@UiO-66	Physical mixing	530 nm (g-CDs) and 375 nm (UiO-66)	1.5–3.5	600–900	Sensing of norfloxacin (NFX)	72
		(*λ*_exc_ = 446 nm)				
HAPNWs-CDs-Tb/MOF	Physical mixing	426 nm (CD)	8.3	—	Sensing of dopamine (DA)	73
		543 nm (Tb-MOF)				
r-CDs@Cu-MOF	Physical mixing	600 nm (r-CDs) and	4 ± 0.31	27 ± 3	Cancer therapy	74
		650 nm (Cu-MOF)				
		(*λ*_exc_ = 520 nm)				
CDs@PCN-224	Physical mixing	520 nm (CD)	2 nm	122.4 ± 2	Cancer therapy	75
		(*λ*_exc_ = 808 nm)				
N-CDs@ZIF-L	Physical mixing	440 nm (N-CD)	—	2000–3000	Antibacterial activity	76
		(*λ*_exc_ = 360 nm)				
		490 nm (N-CD)				
		(*λ*_exc_ = 430 nm)				
Fe-PM@N-CDs	Physical mixing	426 nm (N-CD)	3.4 nm	100	Antibacterial activity	77
		(*λ*_exc_ = 365 nm)				
		630 nm (Fe-PM)				
		(*λ*_exc_ = 365 nm)				
CDs@UiO-66(COOH)2	One-step method	511 nm (CD)	5–8	—	Sensing of chlortetracycline (CTC)	78
		(*λ*_exc_ = 400 nm)				
CDs@ZIF-8	One-step method	450 nm (CD)	1.4–3	200	Sensing of hydrogen peroxide (H_2_O_2_) and glutathione (GSH)	79
		(*λ*_exc_ = 360 nm)				
		560 nm (CD)				
		(*λ*_exc_ = 480 nm)				

It is also possible to enhance the applicability of MOFs by incorporating various types of CDs into the MOF structure. In this way, through the bottle-around-ship method, N. A. Travlou *et al.*^64^ synthesized a hybrid material composed of Ag-MOF and S- and N-doped carbon dots (S-CDs and N-CDs, respectively), which has been applied as an antibacterial agent. To synthesize the S-CDs and N-CDs, poly(sodium 4-styrenesulfonate) and polyvinylpyrrolidone, respectively, were used as precursors. These aqueous solutions were heated in a Teflon-lined autoclave at 200 °C for 6 h. The hybrid compound was prepared by mixing an aqueous solution of trimesic acid (BTC) acid and NaOH (pH 7), where solutions of Ag(NO_3_) and S-CDs/N-CDs were added *via* dropwise addition. FTIR spectra showed that S-CDs primarily contain sulfonate, sulfate, and carboxyl groups, while N-CDs contain amines, amides, and carboxyl groups. Moreover, changes in the IR signals of the carboxylate groups from the MOF were observed, revealing that the functional groups of CDs interact with the metal lattice.

**Fig. 2:**
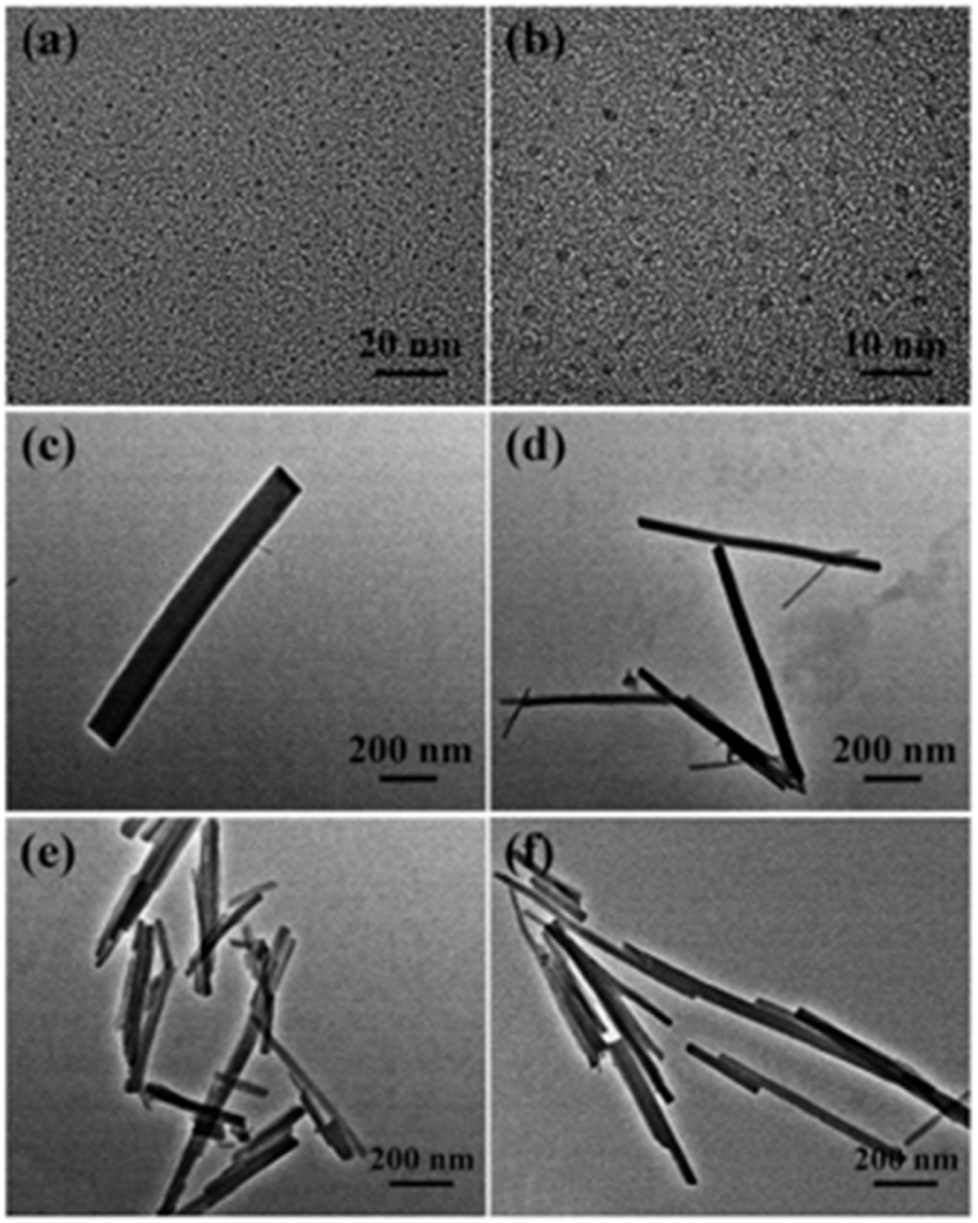
Transmission electron micrographs of carbon dots (a and b), CDs@MOFs(Eu) (c and d), and MOF(Eu) (e and f). Adapted/reproduced from ref. 67 with permission from the Royal Society of Chemistry, Copyright 2018.

On the other hand, it is also possible to obtain a CDs@MOFs composite in which both the CDs and the MOF exhibit optical properties. An example of this is the work of Xin Fu *et al.*,^67^ who designed a dual-emitting hybrid material (CDs@MOFs(Eu)). This compound demonstrated high sensitivity and selectivity for the detection of doxycycline (DOX). The CDs were synthesized using the hydrothermal treatment of citric acid and ethylenediamine. To obtain the hybrid compound, the CDs were incorporated into a mixture with Eu(NO_3_)_3_·6H_2_O and 1,3,5-benzenetricarboxylic acid (H_3_BTC) in a DMF/ethanol solution, and the reaction was carried out at 80 °C for 24 hours. SEM and TEM analyses confirmed the preservation of the rod-like structure of the MOF in the CDs@MOFs composite (Fig. 2). The incorporation of CDs in the MOF was confirmed through XRD, and the presence of relevant functional groups from the CDs was demonstrated using FTIR spectroscopy.

The morphology of the composites was evaluated using TEM. Fig. 2a and b show that the CDs are spherical, with a size of approximately 2 nm, whereas the hybrid material and the MOF(Eu) exhibit a rod-like morphology and comparable dimensions (Fig. 2a–f).

As shown in this section, the bottle-around-ship method offers several notable advantages in the synthesis of CDs@MOFs hybrid materials. The main strength of this method lies in its simplicity and versatility, allowing it to be applied to a wide variety of combinations of CD and MOF structures. Furthermore, this strategy facilitates the efficient encapsulation of CDs in the MOF matrix, preventing their agglomeration on the external surface and promoting a homogeneous distribution throughout the material. Another key factor is that the CDs can be pre-synthesized, allowing their size and morphology to be tailored to the specific requirements of the application. In addition, this approach avoids blocking the MOF pores, maintaining its functional porous structure, and reducing the resistance to nanoparticle diffusion into the material.^80^ However, this method also has certain limitations. During synthesis, metal ions from the MOF precursor can interfere with the CDs, leading to a decrease in their fluorescence intensity.^81^

Moreover, the presence of CDs in the MOF formation stage can negatively affect its crystal growth and optical properties.^82^ Despite these disadvantages, the bottle-around-ship method is promising due to its simplicity and effectiveness in integrating CDs into MOF arrays.

#### 2.1.2. Physical mixing

Another widely used approach for the formation of CDs@MOFs is the “physical mixing” method due to its ease of synthesis. In this case, both the pre-synthesized CDs and the MOF are directly mixed, generating interactions between them, often assisted by binding agents, as shown in the second approach in Fig. 1.^83^

In this context, Pirot *et al.*^71^ reported the development of a hybrid material composed of yellow-emissive CDs (y-CDs), a chromium-based MOF (Cr-MOF), and a molecularly imprinted polymer (MIP), denoted as y-CDs@Cr-MOF/MIP. This composite exhibited high sensitivity for the detection of uric acid (UA) and ascorbic acid (AA) through two distinct recognition sites, enabling its application in real human urine samples. The y-CDs were synthesized using the hydrothermal method using OPD as a precursor at 180 °C for 14 h, while the Cr-MOF (MIL-101(Cr)-NH_2_) was obtained through a hydrothermal method using Cr(NO_3_)_3_·9H_2_O, 2-aminoterephthalic acid, and NaOH in water at 150 °C for 12 h. Subsequently, the composite was prepared by mixing the y-CDs with the Cr-MOF in an aqueous solution under stirring for 5 minutes. After this, the MIP polymer was added by combining AA and UA with (3-aminopropyl)triethoxysilane (APTES) in the mixture, followed by continued stirring. After 2 hours of stirring, TEOS and NH_3_ were introduced, followed by stirring for an additional 16 hours at room temperature. The morphology and crystal structure of y-CDs@Cr-MOF/MIP were analyzed using FE-SEM, revealing that the particles were spherical and polydisperse in size, with average diameters ranging from 25 nm to 788 nm. The resulting nanocomposite probe features two recognition sites and exhibits dual fluorescence, with a blue emission from the Cr-MOF and a yellow emission from the y-CDs.

Another composite, developed by Shixin Wu *et al.*,^72^ is based on green-emitting CDs (g-CDs) and UiO-66 (g-CDs@UiO-66). This material combines the excellent optical properties of g-CDs with the ability of UiO-66 to detect norfloxacin (NFX) in milk and pork samples. The g-CDs were obtained using riboflavin by hydrothermal synthesis in anhydrous ethanol at 180 °C for 10 h after prior sonication. Alternatively, UiO-66 was obtained using solvothermal synthesis in DMF using ZrCl_4_, benzoic acid, *p*-phthalic acid, and trifluoroacetic acid (TFA), with successive reactions at 100 °C for 1 h and 120 °C for 48 h. To synthesize the g-CDs@UiO-66 hybrid material, UiO-66 was dispersed in a solution of g-CDs and stirred for 12 hours at 60 °C.

The integration of the g-CDs into the UiO-66 structure was achieved due to the abundance of functional groups (hydroxyl and carboxyl) on the g-CDs, which can interact with the carboxyl groups of NFX, thus enhancing the fluorescent response. TEM and SEM characterization also confirmed the incorporation of g-CDs into UiO-66. The g-CDs were spherical, well-dispersed, and 1.5–3.5 nm in size, and the UiO-66 maintained its *ortho*-octahedral morphology, with a rougher surface after integration.

An interesting composite based on hydroxyapatite nanowires (HAPNWs) decorated with CDs and a lanthanide metal–organic framework (Tb-MOF) was designed by Mengyao Sun *et al.*^73^ as a ratiometric fluorescent probe for the detection of dopamine (DA). The HAPNWs-CDs-Tb/MOF nanocomposite was prepared through a multi-step process. First, CDs were synthesized using the solvothermal method from glycine in a water/ethylene glycol mixture (15 : 10 mL) at 180 °C for 12 h. In parallel, the Tb-MOF was obtained by mixing Tb(NO_3_)_3_·6H_2_O and trimellitic acid (H_3_BTC) in aqueous and ethanolic solutions, respectively. Subsequently, HAPNWs were synthesized *via* the solvothermal method, onto which the Tb-MOF was grown *in situ*. Finally, the resulting precipitate was dispersed in the CD solution, stirred for 30 min at room temperature, and collected by centrifugation, yielding the composite with CDs homogeneously decorated on its surface through physical mixing.

**Fig. 3:**
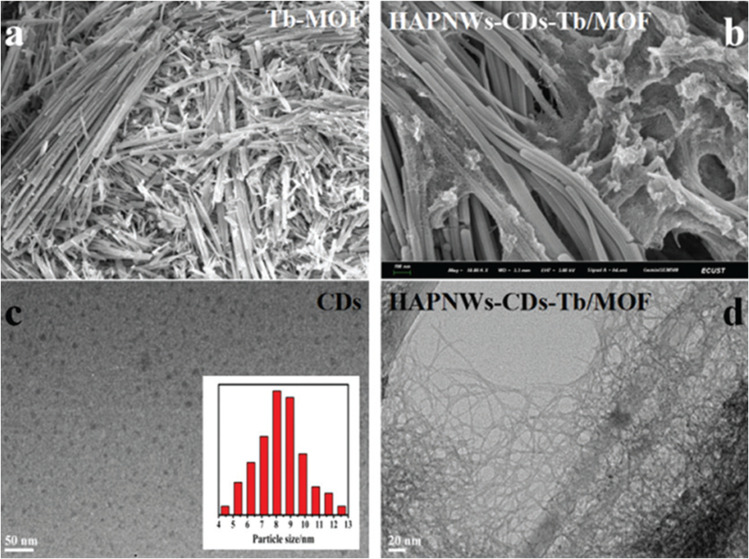
SEM image of HAPNWs (a), SEM image of the HAPNWs-CDs-Tb/MOF (b), TEM image of CDs (c), and TEM image of the HAPNWs-CDs-Tb/MOF (d). Adapted/reproduced from ref. 73 with permission from the Royal Society of Chemistry, Copyright 2022.

SEM images confirmed the integration of the Tb-MOF into the HAPNWs through *in situ* growth, resulting in the characteristic helical morphology, as shown in Fig. 3a and b. The CDs had particle sizes ranging from 4.5 to 12.3 nm, with an average of 8.3 nm (Fig. 3c). The TEM image of the HAPNW-CDs@Tb/MOF showed dense and homogeneous decoration of CDs on the surface of the hydroxyapatite nanowires (Fig. 3d). After the incorporation of Tb-MOF, the surface Tb content increased to 41.01%. SEM-EDS images showed that Tb is uniformly distributed on the surface of the HAPNWs, adopting a helical morphology. Finally, the fluorescence spectrum exhibited the emission characteristics of both the CDs and the Tb-MOF.

On the other hand, materials based on CDs and MOFs have been explored for the development of new cancer therapies. A prominent example is the nanocomposite composed of copper-based MOF nanoparticles (Cu-MOF) doped with red-emitting CDs (r-CDs), denoted as Cu-MOF@r-CD, designed by Zhongping Su *et al.*^74^ In this case, r-CDs were synthesized from OPD through a combined microwave-assisted process at 150 °C for 20 min, followed by hydrothermal treatment at 200 °C for 12 h in the presence of HCl and *tert*-butylhydroperoxide (TBHP). The Cu-MOF was prepared by the controlled addition of Cu(CH_3_COOH)_2_ and H_3_BTC solutions in a DMF/ethanol mixture. Finally, the two components were combined by physical mixing: Cu-MOF and r-CDs were dispersed in ethanol and stirred for 24 h, and the resulting Cu-MOF@ r-CD composite was washed and stored in ethanol at 4 °C.

The Cu-MOF@r-CD nanocomposite showed a lower specific surface area than the Cu-MOF, indicating the successful incorporation of the r-CDs into the pores of the MOF. TEM images confirmed the retention of the original morphology, while Raman spectra revealed an increase in the degree of graphitization of the r-CDs and their successful integration into the MOF. The XRD pattern indicated that the MOF crystal structure remained unchanged after incorporating the r-CDs. Regarding optical properties, Cu-MOF@r-CD retains the characteristics of both components, with a notable increase in near-infrared absorption (660–810 nm), weakening of visible absorption, and redshift in the UV peak of the r-CDs (from 355 nm to 420 nm). This broad absorption in the visible and infrared regions enabled the evaluation of its photothermal performance using an 808 nm laser.

Because the properties of CDs and MOFs can be pre-tuned during their separate synthesis, the physical mixing approach is presented as one of the simplest and most straightforward strategies for obtaining CDs@MOFs composites. CDs can be incorporated into the MOF structure in different ways, either through adsorption on its surface or by occupying the internal pores of its matrix.^84^

Ultimately, the possibility of pre-synthesizing and tuning the characteristics of both components makes physical mixing a versatile and easily adaptable methodology, facilitating the selection and optimization of specific applications for the resulting compounds.

Compounds obtained through this strategy have been used in diverse applications, such as sensing of biomolecules,^71,85–89^ sensing of antibiotics,^72^ biomarker detection,^90,91^ cancer treatment,^74^ and antibacterial applications.^76,92^

### 2.2. One-step method

In the one-step method, as described by the third scheme in Fig. 1, MOFs and CDs are synthesized simultaneously in a single vessel.^49^ To achieve this, MOF precursors, such as metal salts and organic ligands, are mixed with carbon sources and reacted under controlled conditions, resulting in the co-formation of the CDs@MOFs hybrid composite. This approach enables the direct incorporation of CDs into the MOF structure in a single step, thereby obtaining integrated hybrid materials.

The hybrid material CDs@UiO-66(COOH)_2_, developed by Xinlei Zhang *et al.*,^78^ was synthesized using an efficient, solvent-free method for application in the detection of chlortetracycline (CTC). This compound was obtained by dry milling ZrOCl_2_·8H_2_O, pyromellitic acid, and OPD, followed by heat treatment at 180 °C for 24 h. During this process, the OPD molecules were assembled through hydrogen bonding with the MOF and, upon heating, partially decomposed, forming CDs encapsulated within the UiO-66 structure.

**Fig. 4:**
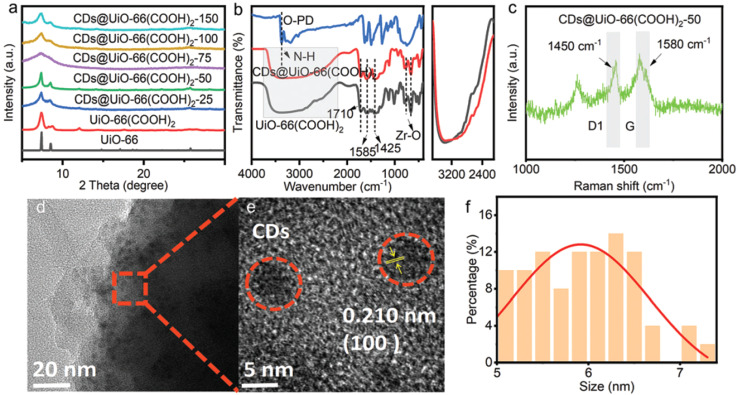
(a) (From bottom to top) Simulated XRD pattern of UiO-66 (JCPDS no. 36-1451), and the experimental PXRD patterns of the as-prepared UiO-66(COOH)_2_ and CDs@UiO-66(COOH)_2_-*X* (*X* = 25, 50, 75, 100, and 150) samples; (b) FTIR spectra of OPD, UiO-66(COOH)_2_ and CDs@UiO-66(COOH)_2_-50; (c) Raman spectrum of CDs@UiO-66(COOH)_2_; and (d) and (e) TEM images and (f) particle size distribution histograms of CDs. Adapted/reproduced from ref. 78 with permission from the Royal Society of Chemistry, Copyright 2022.

The synthesis of the composite was confirmed by various analyses. The XRD patterns of UiO-66(COOH)_2_ and CDs@UiO-66(COOH)_2_ were consistent with that of the simulated crystal, validating its successful production *via* the one-pot synthesis (Fig. 4a). The hydrogen-bonding interactions between the OPD and MOF were confirmed using FTIR analysis, as reflected in characteristic band shifts (Fig. 4b). A homogeneous encapsulation of spherical CDs (5–8 nm) within the MOF crystalline matrix was shown by TEM (Fig. 4d and e). Regarding their PL performance, the CDs@UiO-66(COOH)_2_ composite exhibited intense green emission (around 511 nm) when excited at 400 nm, significantly higher than the weak blue emission of pure UiO-66.

Yufei Wang *et al.* developed a CDs@ZIF-8 nanocomposite using a one-pot method.^79^ The ZIF-8 matrix prevents CD aggregation and quenching, promoting their fluorescence and catalytic activity. The synthesis consisted of grinding ZnO and 2-MeIm in a 1 : 4 ratio, followed by heat treatment at 180 °C for 8 h in a sealed reactor. Structural analyses confirmed the formation of the nanocomposite, with XRD patterns corresponding to standard ZIF-8, but with higher crystallinity. SEM analysis showed spherical crystals for pure ZIF-8 and more irregular particles for the composites. The CDs within the structure of the nanocomposite were evident with a size of ∼1.9 nm. Through XPS and FTIR characterization, C–O and C=O groups attributable to CDs were detected, the content of which varied depending on the synthesis conditions. To corroborate the presence of CDs, they were synthesized separately without ZnO, yielding free CDs with a slightly larger average size of ∼2.4 nm, as shown in Table 1, together with weaker luminescence. This observation demonstrates that ZIF-8 restricts their growth and enhances their fluorescence through quantum confinement.

This methodology allows part of the 2-MeIm to form the ZIF-8 network, while the excess is carbonized to generate CDs, some of which are encapsulated and others removed after washing. Regarding their optical properties, CDs@ZIF-8 exhibited blue-shifted fluorescence (450 nm) relative to pure ZIF-8 (433 nm). Under excitation at 480 nm, an intense yellow emission (560 nm) is observed, which is attributed to the CDs. Furthermore, the incorporation of CDs into ZIF-8 facilitated electron transfer, resulting in high peroxidase-like activity.

The one-step method for synthesizing CDs@MOFs composites is an efficient, rapid, and sustainable strategy as it requires less time, solvents, and energy.^93^ For its success, it is essential that the synthesis conditions allow for the simultaneous formation of MOFs and CDs without one interfering with the crystallization or fluorescence of the other, and that CDs are controlled so that they do not form outside the MOF cavities. However, as the optimal temperatures for synthesizing MOFs are typically lower than those required to carbonize the CD precursors, this method is only viable in specific systems and is not universally applicable, which necessitates further optimization. Compounds obtained using this strategy have been applied in different fields, including biomolecular^79^ and antibiotic sensors.^78^

In summary, the synthetic methodology, particle size, and application of each composite reviewed in this work are shown in Table 1. Although the particle size of CDs@MOFs composites has not been reported in all articles, the reported values cover a broad range, from 25 nm to 3 µm. Larger particle sizes have been obtained *via* the physical mixing method, which can be a drawback for certain applications despite its synthetic simplicity. On the other hand, most composites exhibit fluorescence, which is easily ascribed to CD- or MOF-based luminescence and is determined by the intrinsic nature of each material and not by the synthetic method. However, the chemical, electrochemical, and optical performance are strongly affected by particle size and the different interactions arising from each synthetic approach.

Among the MOFs discussed, ZIF-8 is one of the most frequently reported platforms, forming composites with different CDs that have been applied in the sensing of bioanalytes (BChE, FA, NFZ, H_2_O_2_, GSH) as well as for antibacterial applications. This highlights the versatility of ZIF-8 as a structural host that synergistically enhances material properties.^94^

The various synthetic methods for fabricating CDs@MOFs-based sensors provide researchers with valuable tools for the rational design and synthesis of these materials. Future development should focus on creating hybrid structures with greater precision and incorporating advanced functionalities, such as the inclusion of multiple emitting centers or the addition of nanoparticles. These improvements will significantly expand their applicability in various areas, especially in biosensors and biomedical applications.

It is worth noting that another approach used to obtain hybrid materials is the so-called “ship-in-a-bottle” approach. As shown in the fourth scheme in Fig. 1, this method consists of synthesizing CDs in the presence of the MOF, promoting their formation within the cavities of the scaffold.^52,95^ However, to our knowledge, no CDs@MOFs materials prepared using this strategy have yet been reported for biological applications. The high temperatures and long reaction times required may hinder successful CD formation while compromising the structural stability of the MOF.

## 3. Toxicity and biocompatibility

The assessment of the toxicity and biocompatibility of CDs@MOF materials must account for the combined effects of both components.

MOFs are highly versatile materials with great potential in medical applications, although their toxicity remains a concern. The release of metal ions, such as Zn^2+^ in the case of ZIF-8, and organic ligands during MOF degradation has been shown to cause oxidative stress, mitochondrial dysfunction, and loss of cell viability depending on the size, morphology, stability, and chemical composition of the material.^96^ More recent studies have explored MOFs with alternative metal centers, such as Fe-MOF, Cu-MOF, and Zr-MOF, applied in photodynamic therapy, controlled drug release, and biomolecular detection.^97,98^ However, these systems differ markedly in their toxicological profiles depending on the nature of their central metal ion. In general, Zr-MOFs and Fe-MOFs exhibit greater biocompatibility owing to their chemical stability and low ion release,^99^ while Cu-MOFs and other redox-active metals tend to generate ROS through Fenton-type reactions, increasing the risk of oxidative stress and cell damage.^100^

On the other hand, CDs are highly versatile nanomaterials, but their increasing use in biological applications has highlighted the need to understand their toxic effects. The structural and photochemical stability of CDs is a key factor influencing their toxicity because their degradation or agglomeration can alter the release of reactive species and modify their interaction with cells.^101^ The incorporation of metals, such as Fe, Cu, or Ni, enhances their optical and catalytic properties and increases the generation of ROS, causing oxidative stress, DNA damage, or mitochondrial dysfunction, depending on the type and concentration of the dopant.^102^ Additionally, surface functionalization (introduction of –OH, –NH_2_, or –COOH groups) can improve biocompatibility and reduce cytotoxicity by promoting dispersion and limiting direct interaction with cell membranes.^103^ Taken together, these studies indicate that the toxicity of CDs and M-CDs depends critically on their size, doping, stability, and surface chemistry—factors that must be carefully controlled to ensure their safe use in biological systems.

Regarding CDs@MOF hybrid materials, although there are currently no reviews dedicated exclusively to toxicity, the available experimental results allow the inference of clear trends regarding their biological behavior. Several studies on CDs@MOF hybrid materials, such as those based on ZIF-8, UiO-66, and MIL-101, suggest that the incorporation of CDs can modify the stability, degradation, and cellular response of the MOF, partially reducing its cytotoxicity through surface passivation and decreased release of metal ions. For example, Shuwen Zhou *et al.*^104^ demonstrated that CDs@ZIF-8 exhibited low cytotoxicity (<15% reduction in viability at 100 µg mL^−1^) and notable antioxidant properties in human kidney cells (HK-2), reducing MDA levels and increasing SOD, GPx, and GSH activity by activating the Nrf2/HO-1 pathway. Complementarily, the work by Tian Z. *et al.*^105^ demonstrated that ZIF-8/GQDs exhibited high biocompatibility (cell viability >90% up to 200 µg mL^−1^, cell line 4T1) and pH-dependent drug release, maintaining structural integrity and preventing damage to non-tumor cells. Although GQDs were used in this case, rather than CDs, both nanoforms share a structure based on sp^2^ carbon domains and oxygenated surface functional groups, which explains why their biological interactions and biocompatibility profiles are comparable. These results suggest that incorporating nanocarbons into MOFs can decrease the baseline toxicity of MOFs by stabilizing their structure, modulating the release of metal ions, and improving cellular tolerance. However, in systems containing redox-active metals (Fe, Cu, and Co), CDs or GQDs may promote electron transfer and the formation of ROS, increasing the risk of oxidative stress.^106^

Overall, current evidence indicates that the toxicity of CDs@MOFs depends on the metal composition, CD/MOF ratio, degree of structural integration, and degradation conditions. Therefore, a comprehensive toxicological evaluation is required, including *in vitro* (MTT, CCK-8, and WST-1) and *in vivo* assays, to establish correlations between structure, function, and biocompatibility, thereby determining the viability of these hybrids in biomedical and environmental applications.

## 4. Various applications of CDs@MOFs-based nanomaterials

### 4.1. Biosensing

Biosensing seeks to recognize and quantify analytes using molecular entities that possess high specificity. In this field, as previously mentioned, CDs stand out owing to their intense PL, biocompatibility, and ease of functionalization, enabling the design of optical sensors sensitive to changes in their emission upon interaction with analytes. These sensors can be based on the direct interaction of CDs with the target, their conjugation with specific receptors, or the combination of CDs with other nanocomponents to enhance signal detection.^107^

MOFs, on the other hand, are equally promising due to their porous structure, large surface area, and optical properties, which facilitate analyte accumulation and enhance sensitivity and selectivity upon sensing.^108^ The combination of CDs and MOFs allows the creation of effective hybrid platforms for biological and pharmaceutical detection applications. Recent studies have highlighted that this integration enhances fluorescence responsiveness, improves analyte affinity, and enables more stable and selective biosensing performance owing to the synergistic interaction between the porous MOF framework and the photoluminescent properties of CDs.^109^

Based on the discussion above, the combination of CDs with MOF hybrid materials integrates the advantages of both components. These compounds provide a versatile and tunable platform for detecting various analytes with high sensitivity and specificity.^110^

Depending on the nature of CDs' interaction with analytes, the detection mechanisms can vary. In some cases, CDs serve as primary receptors, while the MOF acts as analyte concentrators, enhancing system sensitivity.^49^ In other approaches, MOFs serve as the primary recognition sites, while fluorescence of CDs is utilized as an internal reference to obtain more stable signals.^111^ There are also designs in which both CDs and MOFs simultaneously participate in analyte recognition, functioning as co-receptors and enabling more precise and quantitative detection.^112^ The latter approach leads to the design of ratiometric fluorescent sensors with dual or multiple emitting centers, showing improved accuracy by minimizing the influence of external factors, such as fluctuations in the excitation source or background absorption.^113^

**Fig. 5:**
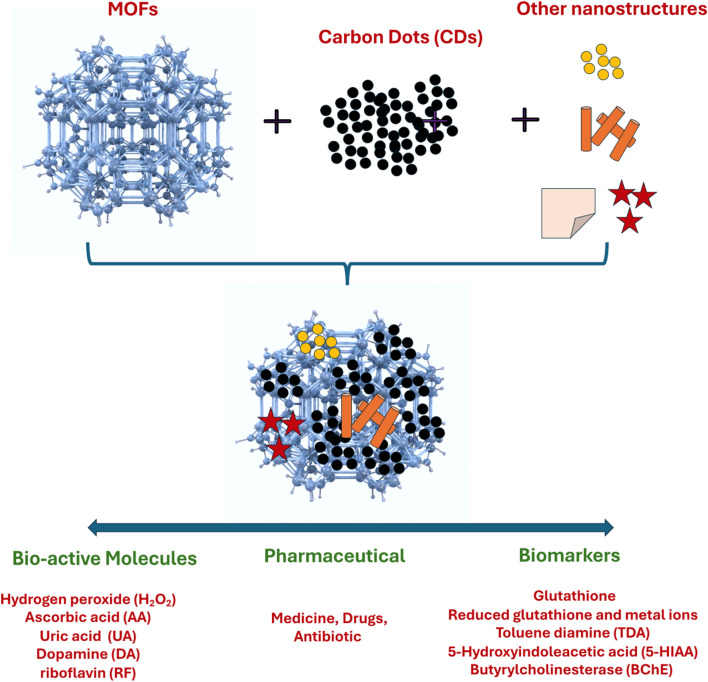
Various CDs@MOFs and other nanocomposites for the sensing of (i) bioactive molecules, (ii) pharmaceuticals, and (iii) biomarkers.

Moreover, the CDs@MOFs hybrid materials can be conjugated with different types of nanomaterials, such as nanoparticles, nanorods, and nanopolymers, to enhance their performances for sensing biomolecules or biomarkers. Fig. 5 summarizes CDs@MOFs and their hybrid materials with other nanocomposites for sensing different types of biomolecules.

**Fig. 6:**
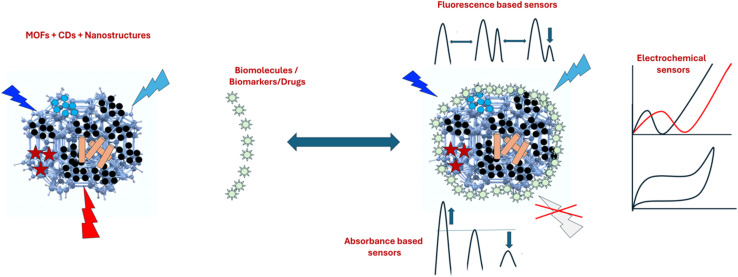
CDs@MOFs and nanostructure-based hybrid materials for the sensing of various biomolecules/biomarkers/drugs by the mechanism of fluorescence/absorbance quenching, signal enhancement, or change in the electrochemical responses.

The underlying mechanisms for the increase or decrease in optical signals (fluorescence, absorbance, or electrochemical) in the presence of an analyte in CDs@MOFs-based systems can be diverse. In this sense, specific interactions lead to different photochemical processes affecting the sensor response. Among the most common fluorescent quenching mechanisms are fluorescence resonance energy transfer (FRET), aggregation-caused quenching, static or dynamic quenching, photoexcitation-induced electron transfer (PET), and the inner filter effect (IFE). These phenomena have been widely studied and discussed in previous work.^114^ This section expands on the discussion of various biosensors for bioactive molecules, drugs, antibiotics, and biomarkers. Fig. 6 shows CDs@MOFs with different nanostructure-based hybrid materials for the sensing of various biomolecules by the mechanism of fluorescence/absorbance quenching, signal enhancement, or change in the electrochemical responses after conjugation with biomolecules.

#### 4.1.1. Bioactive molecules

Some bioactive molecules can diffuse across cell membranes, making it crucial to monitor their concentration.^115^ Below, we present CDs@MOFs-based biosensors designed to detect various bioactive molecules.

##### 4.1.1.1. Hydrogen peroxide (H_2_O_2_)

H_2_O_2_ is a small molecule that can readily diffuse across cell membranes, where it participates in various vital biological processes. In addition to its metabolic roles, it functions as a central signaling mediator in pathways associated with oxidative stress, with H_2_O_2_ often originating from classical enzymatic reactions.^116^ Furthermore, in the bioanalytical field, H_2_O_2_ acts as an intermediate in reactions catalyzed by enzymes, making it an indirect indicator for the detection of biomolecules, including glucose, lactate, and cholesterol (Ch).^117–120^ In this context, evaluating the sensitivity of biosensors to H_2_O_2_ is a classic approach to verify their enzymatic activity. Nanomaterials with enzyme-like properties (nanozymes) have attracted considerable interest as promising platforms for H_2_O_2_ detection, owing to their intrinsic peroxidase-like activity.^121^ These materials can catalyze H_2_O_2_ decomposition or participate in oxidation reactions that generate measurable optical signals, either through fluorescence or color change.^122^ Unlike natural enzymes, nanozymes offer greater stability, lower cost, and resistance to adverse conditions, making them effective alternatives for durable and reusable sensors.^123^ Detection of H_2_O_2_ using nanozymes not only validates their catalytic properties but also expands their applicability in various fields, including biomedicine, clinical diagnostics, environmental monitoring, and food quality control.

CD- and MOF-based materials have demonstrated intrinsic peroxidase-like activity, exhibiting typical nanozyme behavior.^124,125^ Therefore, the development of hybrid CDs@MOFs materials holds great potential for enhanced peroxidase-like performance by synergistically combining the properties of both CDs and MOFs. Yufei Wang *et al.* used a CDs@ZIF-8 hybrid material for the colorimetric detection of H_2_O_2_ and GSH, taking advantage of its notable peroxidase-like activity.^79^ As described in the synthesis section, this material was obtained through a one-step *in situ* hydrothermal method, yielding a compound with optimal catalytic properties suitable for applications in colorimetric sensors. This hybrid material catalyzes the oxidation of the colorimetric substrate 3,3′,5,5′-tetramethylbenzydine (TMB) in the presence of H_2_O_2_, generating a color change from colorless to blue (oxTMB). Regarding the sensing mechanism, kinetic studies were performed using the Michaelis–Menten model with TMB and H_2_O_2_, indicating that photoinduced electrons are transferred from the ZIF-8 LUMO to the CDs, which act as electron acceptors. Electron–hole pair recombination is inhibited as CDs are protected by the ZIF-8 framework, promoting the generation of reactive species from H_2_O_2_ decomposition. Hydroxyl radicals (˙OH) then oxidize TMB, producing oxTMB. Taking this mechanism into account, H_2_O_2_ was detected using UV-vis spectroscopy, where the absorbance at 652 nm was recorded for the quantification of H_2_O_2_. As shown in Table 2, the sensor exhibited a linear detection range of 0.1–1.0 mM for H_2_O_2_ with limits of detection (LOD) of 3.6 µM.

**Table 2:** Biomolecule sensors based on CDs@MOFs hybrid materials

Material	Target	Sensing mechanism	Technique	Linear range	LOD	Real sample	Ref.
CDs@ZIF-8	H_2_O_2_ and GSH	—	Colorimetric	0.1–1.0 mM (H_2_O_2_)	3.6 µM (H_2_O_2_)	—	79
				0–100 µM (GSH)	1.04 µM (GSH)		
CDs@ZIF-CuNC	H_2_O_2_ and UA	IFE	Fluorescence	1–100 µM (H_2_O_2_)	0.30 µM (H_2_O_2_)	Human urine samples	86
				1–30 µM (UA)	0.33 µM (UA)		
CDs@MOF MIL-101(Fe)	H_2_O_2_ and Ch	IFE	Ratiometric fluorescence	2.5–125 µmol per L (H_2_O_2_)	1.354 µmol per L (H_2_O_2_)	Human serum	126
				5–100, 100–1000 µmol per L (Ch)	4.55 µmol per L (Ch)		
N-CDs@Ni-MOF	AA and Fe^3+^	Static quenching	Fluorescence	0.029–8.0 µg per mL (Fe^3+^)	0.0098 µg per mL (Fe^3+^)	Water samples and foods	56
				0.263–18.0 µg per mL (AA)	0.0876 µg per mL (AA)		
B-CuNCs@ZIF-8/y-CDs@MIP	AA	Static quenching	Fluorescence	4.0–22.0 µM	1.56 µM	Drug samples and human serum	88
y-CDs/Cr-MOF@MIPs	UA and AA	Dynamic quenching	Fluorescence	25.0–425.0 µM (AA)	1.30 µM (AA)	Human urine	71
				25.0–425.0 µM (UA)	1.10 µM (UA)		
CDs@ZIF-8	DA	Static quenching	Fluorescence	0.1–200 µM	16.64 nM	Serum and urine	55
HAPNWs-CDs-Tb/MOF	DA	—	Fluorescence	0.04–20 µM	12.26 nM	Human serum	73
CNQDs@Zn-MOF	RF	FRET	Fluorescence	0.005–1.0 µM	15 nM	Milk and vitamin B2 tablets	85
N-CDs@ZIF-67 (NCZ-67)	BSA	FRET	Fluorescence	—	0.2129 µM	Artificial body fluids	127

Another optical sensor for the detection of H_2_O_2_ and UA was developed by Chunran Ma *et al.*,^86^ who designed a ratiometric fluorescent probe based on the CDs@ZIF-CuNC hybrid material by incorporating copper nanoclusters (CuNCs) onto a CDs@MOFs base. This material was synthesized using an *ex situ* method, involving sonication and physical mixing of the components, which enabled the integration of the fluorescent properties of the CDs and the catalytic activity of the CuNCs into a single sensor platform. Two characteristic fluorescent bands were observed upon excitation at 360 nm: one at 460 nm, attributed to the CDs, and another at 620 nm, corresponding to ZIF-CuNC. In the presence of H_2_O_2_, a rapid decrease in fluorescence intensity is observed at 620 nm, while the emission at 460 nm remains practically unchanged. Thus, a ratiometric fluorescent detection strategy was implemented using the signal at 620 nm as the analytical variable and that at 460 nm as the internal reference. Under these conditions, the accurate quantification of H_2_O_2_ was achieved in a linear range of 1–100 µM, with a detection limit of 0.30 µM.

The comparison between CDs@ZIF-CuNC and CDs@ZIF-8 (without copper nanoclusters) revealed significant differences in sensing mechanisms. While the CDs@ZIF-8-based sensor operates through a colorimetric method, the CDs@ZIF-CuNC system employs a ratiometric fluorescent strategy, leveraging the characteristic emission of copper nanoclusters (ZIF-CuNC). This methodological difference affects their analytical performance: the CDs@ZIF-CuNC sensor achieves a lower limit of detection (LOD) of 0.30 µM, which is ten times lower than that obtained using CDs@ZIF-8. However, the colorimetric sensor presents a wider linear working range (0.1–1.0 mM), compared to the 1–100 µM interval achieved by the CDs@ZIF-CuNC system.

On the other hand, in the study by Qiuyu Ye *et al.*,^126^ a ratiometric fluorescent sensor was developed for the detection of H_2_O_2_ and Ch, based on a hybrid material composed of CDs and MIL-101(Fe), which exhibits peroxidase-like activity. The CDs were synthesized *via* the hydrothermal method using citric acid and ethylenediamine at 200 °C for 5 h, while the MIL-101(Fe) was obtained *via* solvothermal synthesis from ferric chloride hexahydrate (FeCl_3_·6H_2_O) and terephthalic acid (H_2_BDC) in dimethylformamide (DMF) at 110 °C for 20 h. Finally, the CDs@MOF nanocomposite was obtained by physical stirring, combining a solution of CDs with MIL-101(Fe) in water, followed by ultrasonic dispersion and magnetic stirring for 48 h. In the presence of H_2_O_2_, this hybrid material catalyzes the oxidation of OPD to 2,3-diaminophenazine (DAP), generating a yellow fluorescent emission at 556 nm. Simultaneously, the intrinsic emission of the CDs at 455 nm is attenuated by the IFE. This variation in the intensity ratio between the two emissions allows the establishment of a reliable ratiometric signal for the quantitative detection of H_2_O_2_. Table 2 summarizes various fluorescence, colorimetric, and ratiometric sensors based on the CDs@MOF and other nanostructures.

##### 4.1.1.2. Ascorbic acid (AA)

AA, commonly known as vitamin C, is a naturally occurring, water-soluble organic molecule present in fresh fruits, vegetables, and fruit juices, and it is also included in numerous multivitamin formulations.^128^ This compound is indispensable for human health because it functions as an antioxidant, coenzyme, free-radical scavenger, and antiviral agent. Owing to its involvement in diverse physiological pathways, AA is fundamental for maintaining normal biological activity.^129^ Insufficient intake of AA may disrupt drug metabolism, weaken the immune defense system, and in severe cases lead to scurvy.^130^ Considering its wide-ranging biological significance, precise quantification of AA is highly relevant in biomedical research because it participates in metabolic processes and has been linked to the prevention or treatment of conditions such as the common cold, infertility, neurodegenerative and neoplastic diseases, and certain HIV-related symptoms.^128^ Furthermore, its protective action against oxidative stress-related disorders, including Parkinson's disease, has been reported.^131^

Ouwen Xu *et al.* developed a fluorescent probe with “on–off–on” behavior based on nitrogen-doped CDs (N-CDs) embedded in a nickel MOF (N-CDs@Ni-MOF), which was obtained by hydrothermal synthesis using the bottle-around-ship method.^56^ The addition of Fe^3+^ ions caused a decrease in fluorescence intensity at the emission peak located at 390 nm related to N-CDs. However, this signal could be recovered after the addition of AA, allowing fluorescence reactivation. This behavior established N-CDs@Ni-MOF as an effective probe for the sequential detection of AA and Fe^3+^ through an “on–off–on” response mechanism. It was proposed that fluorescence quenching is primarily due to a static quenching mechanism, possibly caused by the formation of a non-fluorescent complex in the ground state between the Ni-MOF-N-CDs and the Fe^3+^ ions. As shown in Table 2, with this method, a very low LOD of 0.0876 µg mL^−1^ was obtained in a concentration range of 0.263–18.0 µg mL^−1^, allowing the detection of AA in water samples and foods.

Pirot S. M. *et al.* developed a highly selective fluorescent probe for the detection of AA based on a hybrid material composed of y-CDs, blue copper nanoclusters (b-CuNCs), a ZIF-8-type MOF, and MIP, forming b-CuNCs@ZIF-8/y-CDs@MIP by physical mixing.^88^ The hybrid material incorporates two emitters: CuNCs, which emit at 420 nm and are housed in the pores of the ZIF-8, serving as a stable reference signal, and y-CDs, which emit at 560 nm and are located in the MIP layer, responsible for the detection signal. The integration of an MIP provides highly specific recognition sites for AA, functioning as a synthetic receptor with molecular affinity similar to that of an antibody. Owing to their high selectivity, chemical stability, reusability, and low production cost, MIPs represent an effective alternative to conventional immunoassays in chemical and biomedical sensors. Upon the addition of AA, selective quenching of the yellow signal was observed, attributed to static quenching *via* the formation of a non-fluorescent complex between the fluorophore and AA. In contrast, the blue signal remained constant, serving as an internal reference. This ratiometric sensor showed a linear response in the range of 4.0 to 22.0 µM and a detection limit of 1.56 µM. The probe was successfully applied in the quantification of AA in human serum samples and pharmaceutical products, obtaining satisfactory recoveries.

Similarly, Shano M. Pirot and colleagues developed a fluorescent nanocomposite y-CDs@Cr-MOF/MIP^71^ for the simultaneous detection of AA and UA. As mentioned in the synthesis section, this material was obtained by physical mixing. The resulting system combines the optical properties of y-CDs with the structural advantages of Cr-MOF and the selective recognition capability of the MIP, achieving high specificity toward both analytes.

The hybrid material exhibits well-defined dual fluorescence under excitation at 390 nm: a blue band centered at 450 nm attributed to the Cr-MOF and a yellow emission at 560 nm from the y-CDs. Interaction with AA produces a selective quenching of the yellow signal without affecting the blue fluorescence, while the presence of UA suppresses the blue emission, leaving the yellow emission unaffected. When both analytes are present, both emissions are attenuated. The detection mechanism involves both static and dynamic quenching processes. The probe showed a linear response to AA over a concentration range of 25.0–425.0 µM, reaching a detection limit of 1.30 µM. Furthermore, its applicability to real human urine samples was demonstrated.

##### 4.1.1.3. Uric acid (UA)

UA, chemically referred to as 2,6,8-trihydroxypurine, represents the final metabolic product of purine nucleotides and their derivatives in biological fluids.^132^ Abnormal elevation of UA levels in blood or urine has been linked to numerous pathological conditions and physiological imbalances.^133^ Given its strong clinical significance, precise and sensitive determination of UA is crucial for medical diagnostics and physiological studies.^134^

As mentioned above, Shano M. Pirot *et al.* designed a fluorescent nanocomposite based on y-CDs/Cr-MOF@MIP for simultaneously detecting AA and UA.^71^ This nanocomposite exhibits differentiated fluorescent behavior for each analyte. Thus, when UA is introduced, a selective decrease in the blue emission of the Cr-MOF is observed without affecting the yellow signal of the y-CDs, unlike AA. This differential behavior is due to the specific affinity of each compound for its corresponding recognition site in the MIP matrix, allowing for independent and simultaneous dual analysis. The nanocomposite exhibited a linear response to UA over a concentration range of 25.0–425.0 µM, with a detection limit of 1.10 µM, thereby demonstrating its sensitivity and potential for biomedical applications.

Another example of a fluorescent sensor for UA detection is the previously mentioned spherical CDs@ZIF-CuNC nanocomposite.^86^ This multifunctional material has been used for the detection of H_2_O_2_ and UA. The strategy is based on the use of the enzyme uricase, which catalyzes the oxidation of UA, generating H_2_O_2_ as an intermediate product. This peroxide, produced *in situ*, interacts with the CDs@ZIF-CuNC nanoprobe, enabling the sensitive and specific ratiometric detection of UA. Owing to this enzyme-coupled approach, the probe exhibited a linear response range of 1–30 µM, with a detection limit as low as 0.33 µM. Furthermore, the method was successfully validated using real human urine samples, demonstrating its applicability in clinical settings.

##### 4.1.1.4. Dopamine (DA)

DA is a catecholamine neurotransmitter produced in mammalian nervous tissues and adrenal glands and is predominantly secreted by the hypothalamus and pituitary gland. It exerts a critical influence on numerous physiological pathways, impacting the central nervous, endocrine, and cardiovascular systems. Within the human brain, DA is particularly relevant for regulating movement, mood, motivation, and attention.^135^ Physiological concentrations of DA normally range between 0.01 and 1 µM; deviations from this interval have been correlated with several neurological and psychiatric disorders, including Parkinson's disease, Alzheimer's disease, schizophrenia, attention deficit/hyperactivity disorder, and pituitary tumors.^136^ A reduction in DA often manifests as impaired motor control, whereas excessive DA levels or dysregulation reflect underlying neurological dysfunction. Consequently, the precise detection and highly sensitive quantification of DA are vital for the early diagnosis and clinical monitoring of these conditions, yet remain a considerable analytical challenge in biomedical research.^137^

Siqi Xie and coworkers^55^ developed a fluorescent material by integrating blue-emitting CDs (b-CDs) within the ZIF-8 MOF, thereby obtaining the CDs@ZIF-8 composite using the bottle-around-ship method. This nanoprobe was used for the selective and highly sensitive detection of DA. In this system, the CDs act as fluorescent emitters, converting the chemical signals resulting from interaction with the analyte into detectable optical responses. Owing to the synergy between the CDs and the porous structure of ZIF-8, the detection performance was significantly improved compared with that obtained using individual CDs. The structure of ZIF-8 provides active sites that facilitate the oxidation of DA to dopamine quinone, a species that interacts with the CDs and causes its fluorescence to quench. The DA-induced fluorescence quenching mechanism was investigated in detail. In alkaline solutions, DA is spontaneously oxidized by oxygen, yielding DA quinone, which exhibits a characteristic absorption at around 390 nm. The UV-vis spectra confirmed the formation of these species before and after incubation under basic conditions. As dopamine quinone is a good electron acceptor, interaction with photoexcited CDs induces fluorescence quenching. This interaction is favored by the hydroxyl and carboxyl functional groups on the CD surface, facilitating hydrogen bonding, π–π interactions, and electrostatic forces with the DA functional groups. Hence, DA was quantified through intensity loss of the fluorescent signal upon the addition of the analyte. Time-resolved fluorescence analysis showed that the lifetimes of the ZIF-8 CDs were not significantly altered in the presence of DA, indicating that fluorescence quenching is predominantly static in nature. This system enabled the detection of DA over a wide range of 0.1–200 µM, with a limit of detection (LOD) of 16.64 nM. The applicability of this methodology was successfully validated in real serum and urine samples, demonstrating its feasibility for complex clinical analyses.

Mengyao Sun *et al.* developed an innovative ratiometric fluorescent sensor for DA based on a hybrid material composed of hydroxyapatite nanowires (HAPNWs), CDs, and a terbium-based metal–organic framework (Tb/MOF), denoted as HAPNWs-CDs-Tb/MOF.^73^ This material was obtained by physical mixing, as described in the synthesis section. Distinct from previously reported systems, this synthesis integrates HAPNWs as a structural support, thereby enhancing the stability and overall performance of the sensor.

**Fig. 7:**
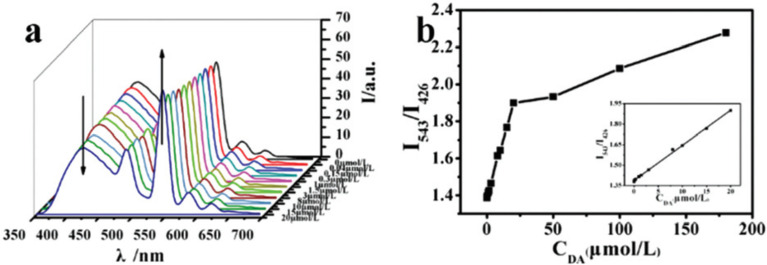
Emission spectra of the HAPNWs-CDs-Tb/MOF solution with different concentrations of DA (a) and fluorescence responses of the HAPNWs-CDs-Tb/MOF solution to various DA concentrations (b) (inset: linear relationship of fluorescence intensity at 543 and 426 nm as the response to the DA concentration). Adapted/reproduced from ref. 73 with permission from the Royal Society of Chemistry, Copyright 2022.

**Fig. 8:**
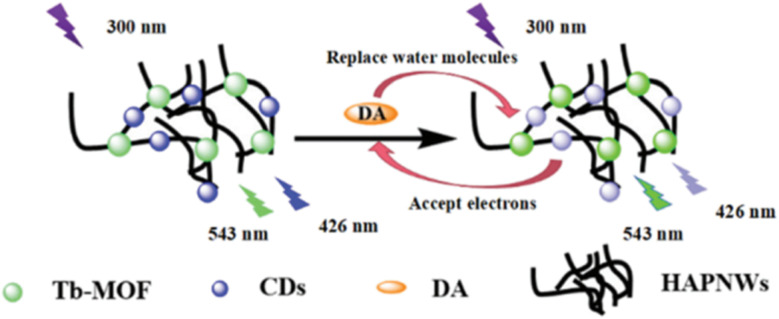
Schematic of DA sensing using HAPNWs-CDs-Tb/MOF as a fluorescent probe. Adapted/reproduced from ref. 73 with permission from the Royal Society of Chemistry, Copyright 2022.

This nanocomposite exhibited dual fluorescent emission: a blue signal at 426 nm, corresponding to the CDs, and a green signal at 543 nm, characteristic of the Tb^3+^ ion (Fig. 7a). These emissions act as the reference and response signals, respectively, for the ratiometric detection of DA. Exposing the probe to increasing concentrations of DA resulted in a progressive decrease in blue emission and an increase in green fluorescence. The loss of CD luminescence is attributed to fluorescence quenching by the analyte, while the coordination of DA to the Tb^3+^ ions, replacing coordinated water molecules, promotes sensitization of the lanthanide-based emission (Fig. 8).

Furthermore, the interaction between the CDs and the quinones altered the recombination kinetics of the electron–hole pairs generated by photon absorption, thereby modifying the fluorescence half-life of the system. The *I*_543_/*I*_426_ intensity ratio increased proportionally with DA concentration, showing excellent linearity in the range of 0.04 to 20 µM (*R*^2^ = 0.9979), within a total detection range of 0–180 µM (Fig. 7b). The detection limit was 12.26 nM. Finally, this method demonstrated its practical applicability when successfully applied to human serum samples, highlighting its potential for biomedical analysis.

##### 4.1.1.5. Other bioactive molecules

Another bioactive molecule of great importance is riboflavin (RF), commonly referred to as vitamin B2, owing to its significant role in energy metabolism and the preservation of cellular health.^138^ Monitoring RF levels is valuable for evaluating nutritional status and ensuring the quality of foods and dietary supplements. Its intrinsic fluorescent properties make it particularly suitable for the design of optical sensing platforms.^139^

In this context, Shasha Feng *et al.*^85^ developed a ratiometric fluorescent sensor for RF detection based on a Förster resonance energy transfer (FRET) system. The sensor utilizes a hybrid material called CNQDs@Zn-MOF, composed of graphitic carbon nitride quantum dots (CNQDs) and a zinc MOF nanocomposite obtained by physical mixing. CNQDs are nanomaterials with a graphitized structure and high nitrogen content, known for their excellent chemical stability, biocompatibility, and optical performance. The material showed excitation-dependent fluorescence from CDs, and irradiation at 365 nm was selected for analytical studies, yielding an emission band at 450 nm with a quantum yield of 35%. The intensity of this band decreases when riboflavin is added, and a new signal is observed at 520 nm. Mechanistical studies indicated that this behavior stems from FRET between the photoexcited CDs and RF acting as an acceptor. Hence, the intensity ratio *I*_520_/*I*_450_ was used to quantify RF concentration, showing a linear dependence. The sensor demonstrated high sensitivity and selectivity toward RF, with a detection limit of 15 nM and a linear response in the range of 0.005–1.0 µM. Furthermore, it was successfully validated in real milk samples and vitamin B2 tablets, demonstrating its applicability in food and pharmaceutical analysis.

As mentioned above, Qiuyu Ye and coworkers^126^ developed a bifunctional ratiometric fluorescent sensor by integrating CDs within the MIL-101(Fe) MOF to quantify H_2_O_2_ and Ch. The importance of this analyte in the clinical field stems from the strong correlation between its blood concentration and cardiovascular risk. For this reason, its precise determination is essential for both preventive strategies and diagnostic purposes.

The nanocomposite is not only luminescent but also exhibits peroxidase-like catalytic activity, as evidenced by the detection of H_2_O_2_. In the presence of H_2_O_2_, the system catalyzes the oxidation of OPD to generate 2,3-diaminophenazine, which emits yellow fluorescence at 556 nm. On the other hand, the intrinsic emission of the CQDs@MOF at 455 nm is attenuated by the internal filter effect. This dual signal modulation enables the intensity ratio to be utilized as an analytical output, thereby constructing a ratiometric biosensor for both H_2_O_2_ and Ch. The method demonstrated high sensitivity and was validated for the accurate determination of total Ch in human serum.

Recently, Maneesha M. *et al.* developed a highly sensitive and selective optical biosensor based on a hybrid composite of nitrogen-doped carbon dots (N-CDs) and the ZIF-67 MOF, termed N-CQD@ZIF-67 (NCZ-67), obtained by physical mixing, for the detection of bovine serum albumin (BSA).^127^ BSA is an important biomarker protein because its concentration in body fluids provides insight into liver and kidney function. Thus, it plays a central role in the diagnosis and clinical monitoring of multiple diseases.^140^ The NCZ-67 sensor demonstrated a remarkable specific fluorescent response to BSA, with a 67.68% signal increase (“on” type), while showing significant quenching efficiencies when exposed to other biologically relevant biomarkers and metal ions, such as creatinine, bilirubin, glucose, urea, UA, glycine, vitamin C, and Na^+^, K^+^, Ca^2+^, Mg^2+^, and Zn^2+^ ions. For evaluation, 5 µM solutions of each analyte were prepared, mixed with the sensor, and stirred for 30 minutes. Their PL spectra were then recorded. The detection mechanism is based on Förster resonance energy transfer (FRET), confirmed by the fluorescence lifetime decay analysis of the material. This sensor exhibited a limit of detection (LOD) of 0.2129 µM and performed well when applied in artificial body fluids, validating its potential for clinical applications in monitoring biomarkers, such as albumin.

#### 4.1.2. Pharmaceuticals

The detection of drugs and antibiotics is crucial in biomedicine, as it ensures therapeutic efficacy, prevents toxic effects, and reduces the risk of bacterial resistance. Furthermore, monitoring these compounds in biological fluids facilitates clinical monitoring and optimization of personalized treatments. In this section, studies related to the use of CDs@MOFs materials for detecting both drugs and antibiotics are discussed.

Quercetin (QCT) is a compound of significant interest because of its strong antioxidant capacity, functioning as an efficient scavenger of free radicals.^141^ Owing to this property, it is considered an important agent in the prevention and management of several diseases, particularly hypertension and certain cancers.^142^ Its therapeutic potential has led to extensive investigation, especially regarding cardiovascular disorders and other conditions linked to oxidative stress.

**Fig. 9:**
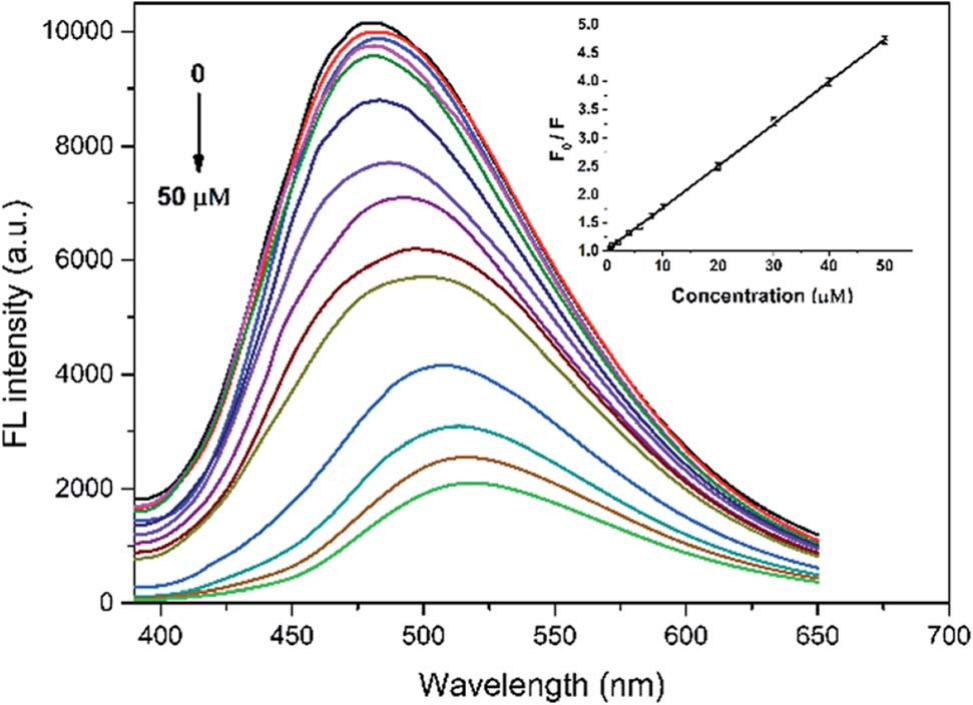
FL emission spectra of CDs@ZIF-8 in the presence of different concentrations of QCT (µM). Adapted/reproduced from ref. 57 with permission from the Royal Society of Chemistry, Copyright 2016.

**Fig. 10:**
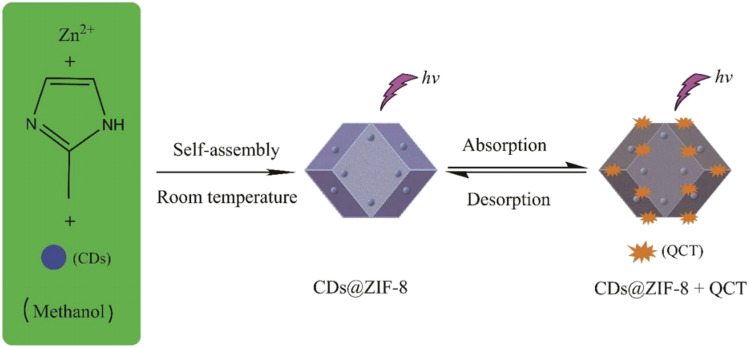
Schematic illustration of the synthesis of CDs@ZIF-8 and its application in QCT sensing. Adapted/reproduced from ref. 57 with permission from the Royal Society of Chemistry, Copyright 2016.

One of the first studies exploring hybrid materials based on CDs and MOF-like structures for fluorescent sensing was conducted by Longhua Xu *et al.*,^57^ who developed a sensor based on the CDs@ZIF-8 composite, synthesized using a simple one-pot route. This system was specifically designed for the detection of quercetin (QCT), achieving high sensitivity with a limit of detection (LOD) of 3.5 nM and a linear quantification range of 0.01–50.0 µM, and was successfully applied to real wine samples. Under 365 nm excitation, the composite emits CD-based fluorescence centered at 480 nm. The progressive incorporation of QCT generates a marked quenching of the fluorescent emission, decreasing the luminescence intensity at 480 nm and redshifting the spectrum. This response was quantified using fluorescence titration assays, where the intensity ratio (*F*_0_/*F*) was related to the QCT concentration, confirming a dose-dependent response over a wide concentration range (Fig. 9). The system performance is attributed to the high affinity of the ZIF-8 framework toward QCT, mediated by π–π interactions and hydrogen bonds. This enables efficient adsorption of the analyte, favoring its accumulation on the sensor surface and enhancing sensitivity. Furthermore, the interaction between the 3-hydroxyl group of QCT and the basic groups on the CD surface generates a stable complex, which facilitates energy transfer and fluorescence quenching. The high selectivity toward QCT is attributed to the synergistic effect between the adsorption capacity of the MOF and the intrinsic sensitivity of the CDs. This sensing approach is depicted in Fig. 10. Comparative tests demonstrated that at the same QCT concentration, the fluorescence intensity of the CDs@ZIF-8 composite remained significantly higher than that of the free CDs, highlighting the superior performance of the composite under identical conditions. This difference corresponds to an improvement in detection capacity when the CDs are embedded within ZIF-8, confirming the crucial role of the hybrid structure in enhancing the performance of the sensor.

Longhua Xu *et al.*^58^ developed a CDs@MOF@MIP-based sensor by combining a MIP with ZIF-8 and CDs, obtained through a one-pot synthesis. The incorporation of the porous MOF in the material leads to higher selectivity and sensitivity toward QCT compared to its non-molecularly imprinted counterpart. Moreover, a faster response was observed, resulting in improved optical detection performance compared to CDs@MIP without MOF. The detection mechanism was attributed to an IFE between CDs@ZIF-8@MIP and QCT. This interaction was confirmed by a fluorescence titration experiment, where a dose-dependent relationship was observed between the fluorescence response (*F*_0_/*F*) and QCT concentration upon the addition of CDs@ZIF-8@MIP to solutions with different analyte concentrations. This sensor was successfully applied to detect traces of QCT, showing a linear decrease in fluorescence over the range of 0 to 50.0 µM, with a detection limit of 2.9 nM. It was also used to quantify the quercetin content in commercial capsules of *Ginkgo biloba* extract.

Jing-Yi Liu *et al.*^66^ developed a hybrid material composed of chiral carbon dots (CCDs) and ZIF-8 (CCDs@ZIF-8), synthesized through a bottle-around-ship method, as described in the synthesis section. The CCDs were obtained hydrothermally using l-cysteine and citric acid as precursors. Owing to their inherent chirality, CCDs are particularly suitable for the detection of chiral compounds. The material was designed as a highly sensitive and selective turn-on fluorescent probe for the detection of d-FA, l-FA, and the antibiotic NFZ. The enantiomers of FA are crucial for cellular metabolism and DNA synthesis, and their distinction is therefore of great significance in biomedical and nutritional research.^143^ NFZ, on the other hand, is a synthetic antibiotic of the nitrofuran class, which has been extensively used to combat bacterial infections; however, its potential toxicity and regulatory controls imposed highlight the importance of its monitoring.^144^ Although individual CCDs showed some ability to detect FA with enantioselectivity, the response of the hybrid system was significantly more precise and linear. Fluorescence lifetime analysis revealed a notable increase upon the incorporation of FA enantiomers, suggesting that chiral matching between the material and analyte suppresses some excited-state distortions that originate from nonradiative relaxation. The LODs obtained were 0.31 µM for d-FA and 4.36 µM for l-FA. In the case of NFZ, it was the only antibiotic that caused a considerable increase in the fluorescence of the system, unlike other antibiotics evaluated, which showed minimal changes. It was shown that NFZ partially affects the structure of ZIF-8, facilitating the release of CCDs and, consequently, enhancing fluorescence emission. Compared to the individual CCDs, whose fluorescence is reduced in the presence of NFZ, the CCDs@ZIF-8 system showed the opposite behavior, possibly due to the synergistic interaction between the CCDs and (2-MeIm). This phenomenon is attributed to a dynamic enhancement mechanism, supported by the reduction in fluorescence lifetime to 1.64 ns upon the addition of NFZ. The detection limit determined for this antibiotic was 3.06 µM. Furthermore, the system proved to be efficient for the detection of analytes in milk samples.

**Fig. 11:**
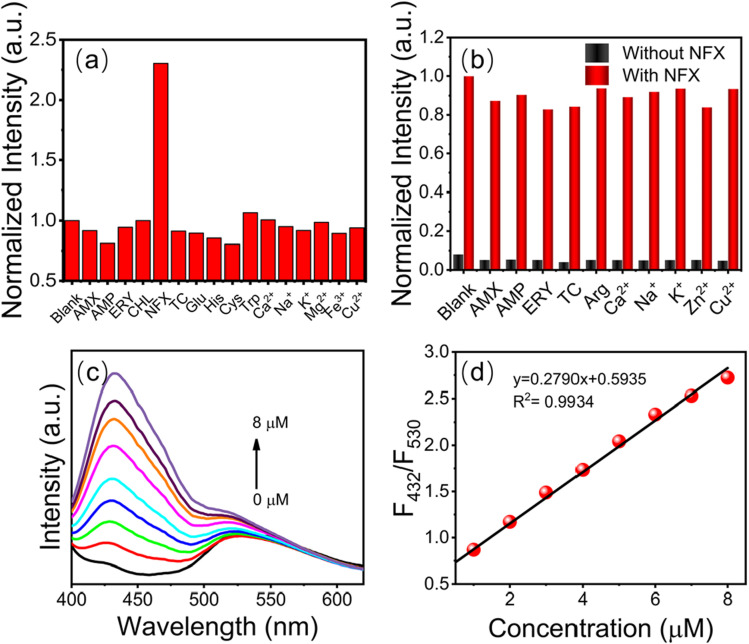
(a) Ratio of fluorescence intensity (*F*/*F*_0_) for g-CDs@UiO-66 in the presence of various antibiotics, metal ions, and amino acids. (*F* and *F*_0_ are the fluorescence intensities at 432 nm in the presence and absence of NFX, respectively). (b) Fluorescence intensity response (*F*/*F*_0_) of g-CDs@UiO-66 to NFX in the presence of different interfering substances (530 nm). (c) FL spectra of g-CDs@UiO-66 at different concentrations of NFX. (d) Linear relationship between *F*_432/530_ and NFX concentration. Adapted/reproduced from ref. 72 with permission from the Royal Society of Chemistry, Copyright 2022.

**Fig. 12:**
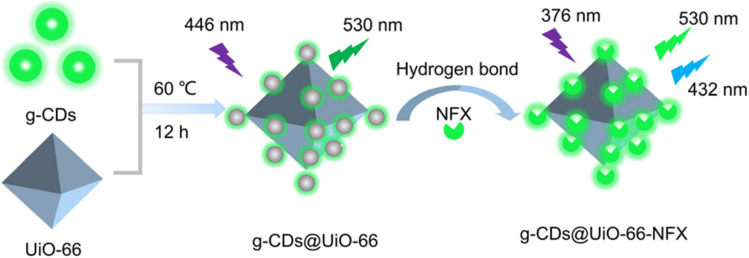
Schematic representation for the preparation of g-CDs@UiO-66 and its applications in NFX sensing. Adapted/reproduced from ref. 72 with permission from the Royal Society of Chemistry, Copyright 2022.

Shixin Wu *et al.* reported the development of a fluorescent composite material, g-CDs@UiO-66, obtained by physically mixing pre-synthesized g-CDs with the UiO-66 MOF. The resulting hybrid was employed as a sensitive probe for the detection of NFX.^72^ NFX is a commonly used fluoroquinolone, which persists in the environment and contributes to antimicrobial resistance, underscoring the importance of its precise detection.^145^ In this work, the g-CDs emit in the green region (536 nm), while UiO-66 emits weakly at 375 nm. On the other hand, NFX presents a weak luminescent band centered at 430 nm. Interestingly, the interaction between g-CDs@UiO-66 and NFX leads to a strong enhancement of NFX fluorescence (Fig. 11c), suggesting low nonradiative rates for this adduct (Fig. 12). Accordingly, the intensity ratio *F*_432_/*F*_530_ was used to quantify NFX at different concentrations, showing a linear correlation in the range of 1–8 mM (Fig. 11d) with a LOD of 0.082 µM. The sensing performance was evaluated with particular attention to its selectivity against common interferents, showing high selectivity for NFX (Fig. 11a and b). Finally, the practical applicability of the sensor was confirmed through successful tests on real milk and pork samples, demonstrating its usefulness for the analysis of NFX in food matrices.

Xin Fu *et al.* synthesized a CDs@MOF(Eu) hybrid material using the bottle-around-ship method, resulting in a dual fluorescent sensor with high sensitivity and selectivity for the detection of DOX.^67^ This analyte, widely used tetracycline antibiotic in both human and veterinary medicine, persists in the environment and contributes to antimicrobial resistance, emphasizing the need for its reliable detection.^146^

This compound exhibited characteristic behavior: as the DOX concentration increased, the blue emission of the CDs (436 nm) decreased, while the red emission of the europium-based MOF (616 nm) intensified. This system exhibited a fluorescent response proportional to DOX concentration, with a logarithmic ratio ln(*F*_616_/*F*_436_) ranging from 0 to 60 µM and a LOD of 0.36 µM, outperforming previously reported methods. The detection mechanism was attributed to two simultaneous processes: FRET between the CD luminescence and DOX absorption and the formation of a DOX-Eu^3+^ complex, which enhances the red emission of the sensor. As a practical innovation, the system was applied for the first time in a “test paper” format, which showed a visible color change from blue-purple to red under UV irradiation (365 nm), facilitating visual detection of the analyte. Furthermore, the material was effective in detecting permanganate (MnO_4_^−^), demonstrating a low detection limit and good tolerance to interferences. The applicability of the sensor was validated in a simulated biological system.

Qingfeng Yang *et al.* synthesized a hybrid material based on nitrogen- and sulfur-doped CDs (NS-CDs) and UiO-67 using the bottle-around-ship method for the recognition of tetracycline (TC),^59^ an antibiotic used in medicine and veterinary practice.^147^ This composite, called NS-CDs@UiO-67, features vertically oriented 2D nanosheets, which enhance mass transport and reduce diffusion barriers, significantly increasing the TC adsorption capacity compared to the original UiO-67. Under initial conditions, the material displays high fluorescence intensity at 410 nm; however, this intensity progressively decreases with increasing TC concentration. This behavior was explained by FTIR, XPS, and zeta-potential studies, which revealed that TC adsorption involves specific interactions with the functional groups of NS-CDs@UiO-67, including hydrogen bonding and π–π interactions between the TC aromatic rings and the MOF ligands. XPS analysis confirmed a reduction in the signal of the Zr–O groups, reinforcing the hypothesis of hydrogen bond formation during adsorption. Additionally, the UV-vis and fluorescence spectra indicated that the TC absorption spectrum overlaps with the NS-CDs@UiO-67 excitation spectrum, evidencing that the sensing mechanism is based on a combination of IFE and FRET, responsible for the observed fluorescent quenching. Under these conditions, the system showed a linear response in the range of 0.08–20.0 mg L^−1^ and a LOD of 0.063 mg L^−1^. The applicability of this sensor was successfully validated in real-world samples of water, fish muscle, and lamb muscle.

Qihui Wang *et al.* developed a hybrid material composed of CDs and a silver-centered MOF (Ag-MOFs), synthesized using the bottle-around-ship method.^60^ This material was used to construct a sensitive and reliable fluorescent sensor for the detection of chloramphenicol (CAP). This antibiotic is banned due to its severe toxicity and may persist as a residue in food and environmental samples, highlighting the need for sensitive detection to ensure safety.^148^ The CDs and Ag-MOFs exhibited emission near 455 and 420 nm, respectively; however, upon the introduction of CAP, a significant quenching of fluorescence was observed at 427 nm under 332 nm excitation. The UV-vis spectra revealed a shift in the absorption band, indicating the formation of a stable complex between CAP and the CD-MOFs. This effect, along with the absence of a shift in the emission wavelength, was attributed to the IFE owing to the overlap between the analyte absorption and the sensor excitation. Quantitative analysis showed that fluorescence progressively decreased with increasing CAP concentration. The Stern–Volmer plot presented an upward curve, suggesting the coexistence of static and dynamic quenching mechanisms. Additionally, transient experiments and theoretical calculations suggested that the dominant mechanism is PET. The sensor achieved a LOD of 44 nM and was validated in real milk powder samples.

Xinlei Zhang *et al.* synthesized a hybrid composite CDs@UiO-66(COOH)_2_ using a solvent-free one-pot method designed for the fluorescent detection of chlortetracycline (CTC).^78^ Extensive use of this antibiotic and its residual persistence raise concerns regarding antimicrobial resistance and public health risks.^149^ In this system, CDs exhibit green emission centered at 511 nm under excitation at 400 nm, while UiO-66(COOH)_2_ displays a very weak blue emission attributed to its organic ligands. During detection, a gradual quenching of fluorescence of the sensor was observed with increasing CTC concentration in a linear range from 0 to 100 µM. The LOD was calculated to be 0.102 µM. Spectroscopic analysis revealed a significant overlap between the UV absorption of CTC and the sensor excitation spectrum, indicating competition for light absorption. However, no overlap was detected between the emission of the sensor and CTC absorption, nor was there any evidence of complex formation between the two, according to the UV spectra in solution. On the other hand, involvement of FRET was ruled out, according to the negligible variation of photoluminescence lifetime upon addition of CTC (1.54 to 1.62 ns). Therefore, the observed fluorescence quenching is primarily attributed to the IFE, identified as the mechanism responsible for the behavior of the sensor with CTC.

#### 4.1.3. Biomarkers

The detection of biomarkers plays a pivotal role in biomedicine, as it supports early diagnosis, continuous disease monitoring, and evaluation of treatment outcomes.^150^ Within this framework, CDs@MOFs-based hybrid materials emerge as promising candidates because their structural and functional features can be tailored to create highly sensitive and selective platforms for biomedical applications. Recently, CQD@MOF hybrids have also been applied to oral disease diagnostics, enabling sensitive and noninvasive biomarker detection and demonstrating their versatility beyond traditional biomedical applications.^151^

##### 4.1.3.1. Glutathione (GSH)

GSH is a crucial intracellular antioxidant that plays a central role in neutralizing free radicals, safeguarding cells from oxidative stress, and regulating key biological processes.^152^ Monitoring GSH levels is highly relevant, as its concentration reflects the cellular redox state; diminished levels indicate oxidative stress or cellular damage, while its imbalance is associated with diseases such as cancer, diabetes, and Parkinson's disease. This makes GSH a valuable clinical biomarker.^153^

As previously mentioned, Yufei Wang *et al.* developed a CDs@ZIF-8 hybrid material for the colorimetric detection of GSH.^79^ In this system, the measurement is performed in the presence of H_2_O_2_ and TMB, taking advantage of the catalytic capacity of the nanocomposite, which yields oxTMB. Subsequently, upon the addition of GSH, the oxTMB is reduced, resulting in a decrease in absorbance and a color change from blue to colorless in the mixture. The quantitative detection of GSH was performed using UV-vis spectroscopy, recording the absorbance at 652 nm while maintaining a fixed concentration of H_2_O_2_ and TMB. Using this method, they obtained a LOD of 1.04 µM over a concentration range of 0 to 100 µM.

Another colorimetric sensor for detecting GSH was proposed by Jun Zhao *et al.*,^89^ utilizing TMB as a chromophore. In this interesting study, the authors employed a hybrid material composed of N-CDs and the UiO-66 MOF, forming the N-CDs@UiO-66 system, which acts as a nanoenzyme capable of modulating redox balance. The material was obtained through an *ex situ* method by physical mixing, which effectively combined the catalytic properties of the N-CDs with the porous structure of the MOF, facilitating the oxidation of TMB and its subsequent modulation in the presence of GSH. The performance of the N-CDs@UiO-66 nanocomposite in eliminating and generating ROS and free radicals was evaluated. Experimental tests showed that this nanocomposite exhibits activity similar to that of the enzyme superoxide dismutase, along with significant antioxidant properties, as they are capable of neutralizing endogenous free radicals (˙OH, O_2_˙^−^, and ABTS˙^+^). Furthermore, this material is capable of generating ROS (˙OH, O_2_˙^−^, and ^1^O_2_) when exposed to light irradiation, indicating light-induced oxidase-like activity and a pro-oxidant effect. Owing to its irradiation-induced oxidase-like activity, the N-CDs@UiO-66 nanocomposite was used for the detection of Fe^3+^ and GSH. Specifically, colorimetric detection of GSH was performed in the presence of the substrate TMB, following a mechanism similar to that reported by Yufei Wang *et al.*^79^ In this system, N-CDs@UiO-66 catalyzes the oxidation of TMB under irradiation, leading to the formation of a blue product. UV-vis spectroscopy revealed a characteristic absorption peak at 652 nm, confirming the generation of oxidized TMB (oxTMB). The incorporation of GSH was observed to inhibit this oxidation in a concentration-dependent manner. With increasing GSH content, the absorption intensity gradually decreased, reflecting an inhibition proportional to the level of GSH present. A linear relationship between absorbance and GSH concentration was observed in the 0–15 µM range, with a calculated detection limit of 0.48 µM.

Fluorescent GSH sensors have also been reported. Roghayeh Jalili *et al.* developed by-CDs@ZIF-8, a fluorescent ratiometric probe for detecting GSH.^54^ This hybrid material was obtained using the bottle-around-ship method through an *ex situ* process. This material is based on the encapsulation of two types of CDs in ZIF-8: y-CDs, which emit yellow light, and b-CDs, emitting blue light. In this way, the system shows emissions at 565 and 440 nm under excitation at 365 nm. In the presence of Cu^2+^, the yellow fluorescence is intensified, while the blue fluorescence is attenuated. However, upon the addition of GSH, both signals are restored due to its strong interaction with Cu^2+^. The color change from yellow to blue is visible to the naked eye under UV light. Hence, the observed decrease in fluorescence is attributed to a static quenching mechanism. The absorption band of the Cu_24_-y-CDs complex exhibits a notable spectral overlap with the fluorescent emission of the b-CDs in the by-CDs@ZIF-8-Cu system, suggesting the possibility of a FRET process from the b-CDs to the Cu_24_-y-CD complex. This phenomenon would explain the significant decrease in the fluorescence intensity of the b-CDs. This sensor showed a detection range of 3–25 nm and a detection limit of 0.90 nm. Furthermore, the compound exhibits good selectivity, analyte accumulation capacity, and a ratiometric strategy that improves precision. This method was successfully applied to detect GSH in fruit samples.

Jinli Fu *et al.*^87^ developed a hybrid material based on CDs@ZIF-8 for the fluorescence detection of reduced GSH and metal ions. The nanocomposite exhibited enhanced fluorescence with triple emission under dual excitation at 370 and 790 nm, allowing for the simultaneous detection of both analytes. The presence of Fe^3+^ ions suppresses the fluorescence signal due to charge transfer with functional groups of CDs@ZIF-8, which can be restored upon the addition of GSH. For the detection of the GSH, the Fe^3+^@CDs/MIM-Zn system was used, with fluorescence recovery observed in the 400–600 nm range under excitation at 370 nm. This system demonstrated high sensitivity, with an LOD as low as 15 nM, within a linear concentration range of 0.5–70 µM, and was applicable to real, diluted human serum samples.

##### 4.1.3.2. Other biomarkers

Anthrax is a severe infectious disease caused by *Bacillus anthracis*, whose spores exhibit remarkable resistance, ease of dissemination, and significant public health risks, particularly due to their potential misuse in bioterrorism.^154^ Within this framework, calcium dipicolinic acid (CaDPA) and dipicolinic acid (DPA) have emerged as distinctive and selective biomarkers for the identification of these spores.^155^

Kuiyu Yi *et al.* designed a CDs@Eu-MOFs material using a simple one-pot, *in situ* self-assembly synthetic strategy.^61^ In this work, they successfully fabricated a sensitive and selective dual-emission ratiometric fluorescent probe for the detection of CaDPA, a characteristic biomarker of anthrax spores, in both water and human serum samples. In this system, b-CDs (455 nm) act as a stable internal fluorescent reference, as CaDPA does not affect their emission. Meanwhile, Eu-MOFs exhibit Eu^3+^ characteristic red fluorescence signals (592, 616, and 693 nm), which gradually intensified as the CaDPA concentration increased, especially at 616 nm. The intensity ratio (*I*_616_/*I*_455_) exhibits a linear relationship with the CaDPA concentration in the range of 8–170 µg L^−1^, with a detection limit of 0.66 µg L^−1^. The detection mechanism is attributed to CaDPA acting as a sensitizer to the intrinsic luminescence of Eu^3+^ through energy transfer from CaDPA itself to the Eu^3+^ ion. This process is favored by the ability of CaDPA to act as a tridentate chelating ligand owing to its two carboxyl groups and a pyridine nitrogen as a coordination site, allowing a strong metal–ligand interaction and a cation–π interaction with Eu^3+^, leading to a significant increase in Eu^3+^ luminescence.

Another example of ratiometric detection of DPA is the study by Jian Bao *et al.*,^90^ who synthesized the 2D CDs@MOFs hybrid material, where the MOF corresponds to Zn-TCPP, composed of a Zn metal center and a porphyrin ligand (TCPP), obtained by physical mixing. In this system, the CDs emit at 445 nm, while the Zn-TCPP emits at 613 and 659 nm. The fluorescence intensity at 659 nm increased as a result of the release of the TCPP ligands, induced by the selective interaction between DPA and the Zn^2+^ ions of the MOF. Similar to the aforementioned sensor by Kuiyu Yi *et al.*,^61^ the CDs offered a stable reference signal at 445 nm, enabling ratiometric detection with self-correcting capability. The *F*_659_/*F*_445_ ratio showed good linearity with DPA concentrations in the ranges of 0.01–0.2 µM and 0.2–10 µM, under excitation at 390 nm, with a LOD of 7 nM. This sensor was evaluated in fortified biological samples, confirming its applicability.

Toluene diamine (TDA) is not an endogenous metabolite but serves as a biomarker of occupational exposure to toluene diisocyanate (TDI), as TDI is metabolized to TDA, which can be detected in biological samples.^156^ In this sense, Si-Jia Qin *et al.*^62^ developed a dual-emitting hybrid material using the bottle-around-ship method, incorporating CDs and Eu^3+^ lanthanide ions into the MIL-53 MOF, resulting in the formation of the Eu^3+^/CDs@MIL-53 composite. This structure serves as a hybrid ratiometric fluorescent probe, exhibiting high potential as a bifunctional sensor, notable for its high selectivity and sensitivity in detecting TDA, a metabolite of toluene diisocyanate (TDI). In this system, the ratio of fluorescence intensities (*I*_CDs_/*I*_Eu_) increased proportionally with TDA concentration. The ratio of emissions at 440 nm (CDs) and 616 nm (Eu^3+^) was plotted as a function of TDA concentrations, normalized to a solution without TDA. Good linearity was obtained in the calibration curve, and the LOD was determined to be 6.8 µg mL^−1^.

5-Hydroxyindoleacetic acid (5-HIAA), the main metabolite of serotonin (5-hydroxytryptamine), is excreted in urine and serves as an indicator of the homeostatic state of the body.^157^ Its quantification holds considerable clinical importance, as it functions as a specific biomarker for carcinoid tumors and provides valuable information on serotonin metabolism, which is essential for the diagnosis of both neuroendocrine disorders and mood-related conditions.^158^

Based on this application, YuLang Fei *et al.*^91^ designed the b-CDs@EuBTC hybrid material obtained by physical mixing synthesis, composed of b-CDs (*λ*_em_ = 441 nm) and a red-emitting MOF (EuBTC) with peaks at 594 and 616 nm. This dual-emission fluorescence platform was used for the ratiometric detection of 5-HIAA. The detection mechanism is based on the displacement and release of b-CDs from the b-CDs@EuBTC structure by 5-HIAA, causing an increase in blue emission. Simultaneously, the red emission of EuBTC decreases due to thermal relaxation induced by 5-HIAA, thus generating a ratiometric signal dependent on the analyte concentration. This system showed a LOD of 0.3 µM, with a working range of 0.3–70 µM, and high selectivity against other species. Furthermore, the performance of this sensor was validated through its application in real human urine samples, demonstrating its effectiveness as an analytical tool for clinical 5-HIAA monitoring. Moreover, BChE is a plasma enzyme widely recognized as an important biomarker for the diagnosis of liver disorders, which has driven the development of more effective strategies for its detection.^159^ In this regard, Kelin Hu *et al.*^65^ developed a fluorescent sensor based on the CDs@ZIF-8 hybrid material obtained through the synthetic strategy known as bottle-around-ship. This system involves the encapsulation of CDs emitting at 436 nm within a ZIF-8 matrix, which initially quenches the fluorescence of the CDs. However, in the presence of BChE, a competitive interaction occurs between the enzyme and the Zn^2+^ metal ions of the ZIF-8 framework, inducing partial disintegration of the structure and allowing the release of the CDs, restoring their light emission. The detection mechanism was validated using various assays, including the use of various luminescent hosts, metallic nodes, and organic ligands. These studies demonstrated that the electrostatic attraction between BChE and the Zn^2+^ metal centers drives ZIF-8 cleavage. This platform demonstrated high sensitivity, reaching a detection limit of 1.5 U L^−1^, and was successfully evaluated in 40 clinical human serum samples from patients with liver disease, showing results consistent with established medical diagnoses.

The analytical parameters for the sensing of biomolecules, medicaments, and biomarkers are summarized in Tables 2–4, respectively. A great diversity of sensors for different bioanalytes has been developed, showing notable performance, even in real biological samples. In all cases, the sensing methodology is based on optical techniques such as fluorescence and absorbance (colorimetric). For most sensors, the CD-based fluorescence corresponds to the analytical signal, which varies with the analyte concentration. In addition, MOF plays a structural role and contributes to cavities and pores with specific chemical interactions. The synergy between the CD and MOF structures modulates their ability to establish an adduct with other molecules. Furthermore, in some cases, MOF shows luminescence, which can be used for ratiometric sensing when combined with emission from CD. Most studies shed light on the mechanism behind luminescence quenching/enhancing based on spectroscopic analyses (luminescence lifetime or other time-resolved techniques). Hence, different sensing mechanisms are employed such as static quenching and dynamic processes including FRET, IFE, and PET, which can be designed for the specific analyte by choosing an appropriate CDs@MOFs composite.

**Table 3:** Antibiotic and drug sensors based on CDs@MOFs hybrid materials

Material	Target	Sensing mechanism	Technique	Linear range	LOD	Real sample	Ref.
CDs@ZIF-8	QCT	—	Fluorescence	0.01–50.0 µM	3.5 nM	Real wine sample	57
CDs@ZIF-8/MIP	QCT	IFE	Fluorescence optosensing	0–50.0 µM	2.9 nM	Real Ginkgo biloba extract capsules	58
CCDs/ZIF-8	Folic acid and nitrofurazone	—	Fluorescence	—	0.31 µM (FA)	Milk solution	66
					4.36 µM (l-FA)		
					3.06 µM (NFZ)		
g-CDs@UiO-66	Norfloxacin	—	Ratiometric fluorescence	1–8 mM	0.082 µM	Real milk and pork samples	72
CDs@MOF(Eu)	Doxycycline MnO_4_^−^	FRET	Ratiometric fluorescence	0–60 µM	0.36 µM	Simulated biological system	67
UiO-67@NSC	Tetracycline	IFE and FRET	Fluorescence	0.08–20.0 mg L^−1^	0.063 mg L^−1^	Water, fish muscle, and sheep muscle samples	59
CD-MOFs@ Ag-MOFs	Chloramphenicol	PET	Fluorescence	—	44 µM	Milk powder sample	60
CDs@UiO-66(COOH)_2_	Chlortetracycline	IFE	Fluorescence	0–100 µM	0.102 µM	—	78

**Table 4:** Biomarker sensors based on CDs@MOFs hybrid materials

Material	Target	Technique	Linear range	LOD	Real sample	Ref.
N-CDs@UiO-66	GSH and Fe^3+^	Colorimetric	0–15 µM	0.48 µM	—	89
by-CDs@ZIF-8	GSH	Fluorescence	3–25 nM	0.90 nM	Grapes and cucumbers	54
CDs@ZIF-8	GSH	Fluorescence	0.5–70 µM	15 nM	Diluted human serum	87
CDs@Eu-MOFs	CaDPA	Ratiometric fluorescence	8–170 µg L^−1^	0.66 µg L^−1^	Water and human serum	61
CDs@Zn-TCPP	DPA	Ratiometric fluorescence	1.1–0.2 µM	7 nM	Spiked biological samples	90
			0.2–10 µM			
Eu^3+^/CDs@MIL-53	TDA	Ratiometric fluorescence	—	6.8–1 µg mL	—	62
b-CDs@EuBTC	5-HIAA	Fluorescence	0.3–70 µM	0.3 µM	Urine sample	91
CDs@ZIF-8	BChE	Fluorescence	5.0–160.0 U L^−1^	1.5 U L^−1^	Serum sample	65

### 4.2. Cancer detection, diagnosis, and therapy

Cancer is one of the most serious diseases affecting human health, which has stimulated an intense search for more effective methods for its early diagnosis and treatment. In this context, MOFs and CDs have demonstrated promising properties for the development of new therapeutic and diagnostic strategies.^160^ However, CDs@MOFs-based composites have been poorly explored, despite their great potential in this field. This section analyzes the most recent advances and discoveries regarding the use of CDs@MOFs composites in applications related to cancer detection, diagnosis, and treatment.

CDs@MOFs-based nanocomposites can be conjugated with anticancer drugs such as doxorubicin (DOX) or andrographolide (ADG), as well as with specific cancer biomarkers including Human Epidermal Growth Factor Receptor 2 (HER2), Estrogen Receptor (ER), Progesterone Receptor (PR), nuclear proliferation protein (Ki-67), Cancer Antigen 125 (CA-125), Human Epididymis Protein 4 (HE4), and 5-hydroxyindoleacetic acid (5-HIAA). These hybrid systems can selectively recognize and bind to cancer cells, enabling highly sensitive detection, bioimaging, and spatial mapping of tumor regions through multiple analytical techniques, such as PL, ratiometric photoluminescence (rPL), electrochemical detection (EC) (including electrochemical impedance spectroscopy (EIS), cyclic voltammetry (CV), and differential pulse voltammetry (DPV)), colorimetric detection (COL), and mass spectrometry or spectrometric detection (MS/D).

Once accumulated near cancerous tissues, these nanocomposites can respond to external stimuli, such as light, ultrasound, and magnetic fields, to trigger controlled drug release or local heating, thereby promoting selective cancer cell destruction. Depending on the type of activation, several therapeutic modalities can be achieved, including photodynamic therapy (PDT), photothermal therapy (PTT), chemodynamic therapy (CDT), drug/gene delivery (DG), immune checkpoint blockade (ICB), and drug delivery systems (DDSs).

**Fig. 13:**
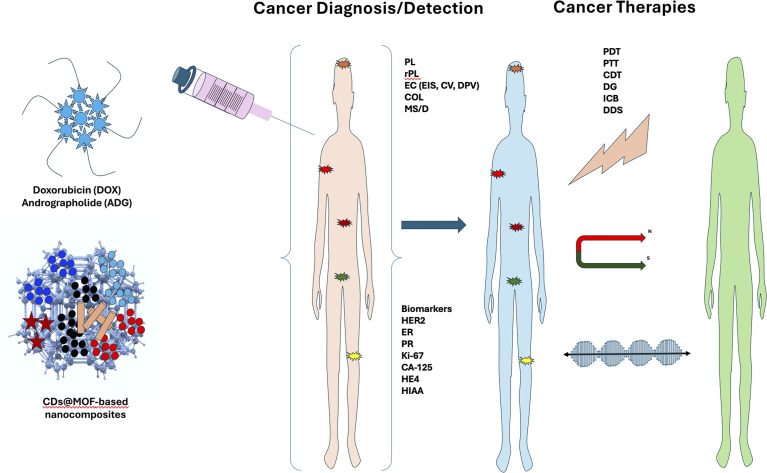
Schematic illustration of CDs@MOF-based nanocomposites for cancer diagnosis/detection and therapy. These hybrid systems can bind anticancer drugs or biomarkers to enable efficient detection and targeted drug delivery. Upon external stimulation, such as light, ultrasound, or other therapeutic triggers, the drugs can be released effectively, resulting in the selective destruction of cancer cells.

As illustrated in Fig. 13, these mechanisms highlight the theranostic potential of CDs@MOFs-based nanocomposites, which integrate diagnostic and therapeutic functionalities within a single platform.

#### 4.2.1. Cancer detection and diagnosis

Early cancer diagnosis is essential to improve patient survival. In this context, biomarkers such as HER2, associated with breast cancer, have gained significant relevance, particularly for breast cancer diagnosis, enabling more precise detection strategies.^161^

Optical and electrochemical sensors have emerged as promising tools due to their high sensitivity, specificity, and potential for miniaturization. While optical sensors leverage properties such as fluorescence and colorimetry to generate visible signals, electrochemical sensors enable rapid, low-cost detection with high quantification capacity. Several studies have explored the use of CDs^162^ and MOFs^16^ to improve the performance of these platforms, with some reports based on the CDs@MOFs composite.^68,163,164^ In this sense, in these types of applications, MOFs provide high porosity, surface area, and functionalization capacity, while CDs offer excellent PL, conductivity, and biocompatibility. The integration of both into the detection platforms enables the design of dual optical and electrochemical sensors capable of simultaneously detecting biomarkers and live cancer cells, significantly improving diagnostic accuracy and opening up new possibilities for noninvasive cancer monitoring.

In an innovative approach for the ultrasensitive detection of cancer biomarkers, Chenxi Gu *et al.* developed a fluorescent electrochemical sensor using a hybrid material composed of CDs embedded in a MOF of zirconium and hafnium (CDs@ZrHf-MOF).^163^ The composite was synthesized by solvothermal treatment at 120 °C using a bottle-around-ship method with previously prepared CDs. During the formation of the MOF, the CDs were encapsulated within the internal cavities without affecting their nanoporous structure. This integration provided high fluorescence properties and abundant surface amino groups.

**Fig. 14:**
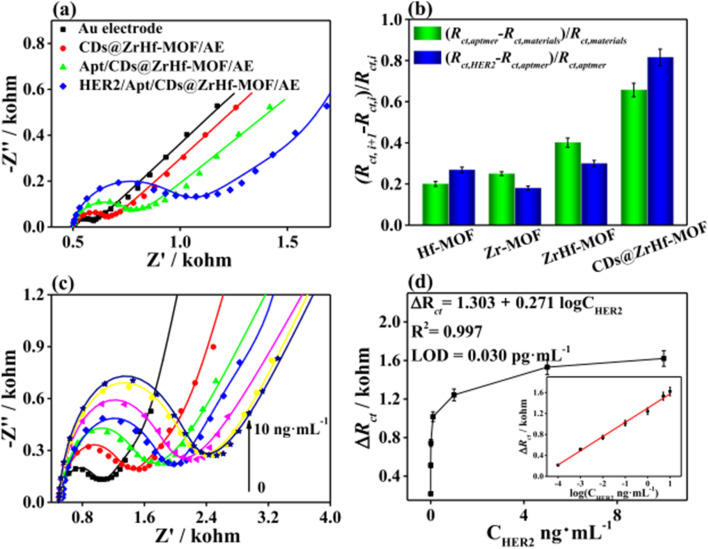
(a) EIS Nyquist plots of the CDs@ZrHf-MOF-modified AE for the detection of 0.0001 ng per mL HER2. (b) Variation in the charge-transfer resistance (*R*_ct_) values for each stage of HER2 detection. (c) EIS responses of the CDs@ZrHf-MOF/AE with different HER2 concentrations (0, 0.0001, 0.001, 0.01, 0.1, 1, 5, and 10 ng mL^−1^). (d) Dependence of Δ*R*_ct_ on the concentration of HER2 (inset: linear parts of calibration curves). Adapted/reproduced from ref. 163 with permission from Elsevier, Copyright 2019.

For cancer biomarker detection, the CDs@ZrHf-MOF nanocomposite was dispersed in water and deposited onto a gold electrode to construct the sensing platform, which was then functionalized with a specific aptamer targeting the HER2 receptor. The CDs@ZrHf-MOF material exhibited a remarkable ability to anchor the aptamer chains, attributed to the protonation of the –NH_2_ groups, which enabled strong electrostatic interactions with the negatively charged phosphate backbone of DNA. This stable immobilization, supported by π–π stacking, hydrogen bonding, and electrostatic forces, facilitated efficient recognition of the HER2 protein and HER2-overexpressing MCF-7 cells. The EIS results (Fig. 14 a–d) confirm the efficient construction and excellent sensing behavior of the CDs@ZrHf-MOF aptasensor toward HER2. As shown in Fig. 14a and b, the progressive increase in charge-transfer resistance (*R*_ct_) verifies each modification step and highlights the improved conductivity provided by the CDs. Upon increasing the HER2 concentration (Fig. 14c), the Nyquist semicircle expands due to aptamer–antigen binding, while the calibration curve (Fig. 14d) exhibits a linear relationship (*R*^2^ = 0.997), with a LOD of 0.030 pg mL^−1^. The incorporation of amino-doped CDs further enhanced aptamer binding, reduced the crystallinity of the MOF, and improved the charge-transfer efficiency, resulting in a sensor with lower *R*_ct_ and superior electrochemical response. Analytically, the aptasensor achieved ultralow detection limits of 19 fg mL^−1^ for HER2 (within 0.001–10 ng mL^−1^) and 23 cells per mL for MCF-7 cells (within 10^2^–10^5^ cells per mL), demonstrating excellent sensitivity, selectivity, reproducibility, and stability for at least 15 days.

From a diagnostic perspective, the CDs@ZrHf-MOF-based aptasensor is a promising tool for early breast cancer diagnosis as it enables the simultaneous detection of circulating tumor markers (HER2) and viable cancer cells. The hybrid structure effectively combines the biocompatibility and bioaffinity of ZrHf-MOFs with the conductivity and fluorescence of CDs, thereby enhancing signal transduction and analytical stability. This multifunctional integration enables accurate, minimally invasive clinical diagnosis and provides a highly sensitive platform for early-stage detection of malignancies.

**Fig. 15:**
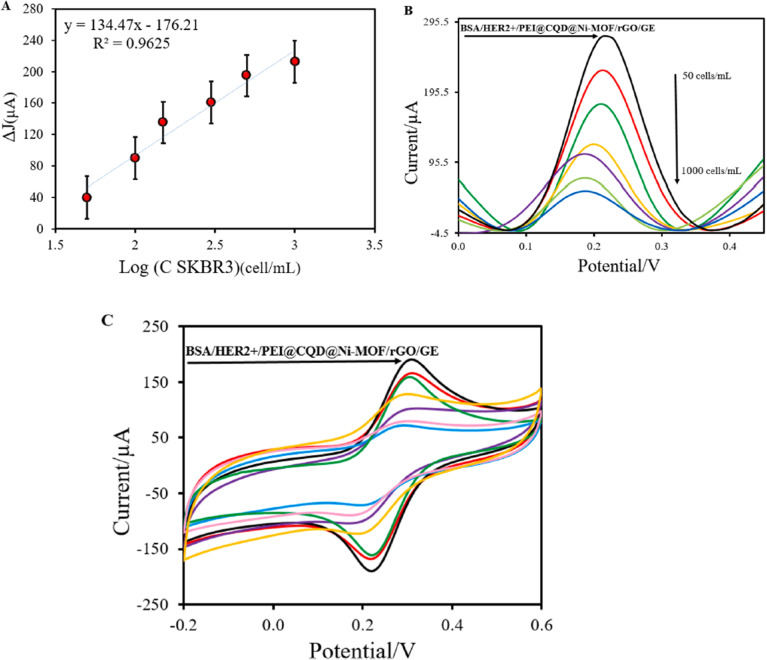
(A) Calibration curve for various numbers of SK-BR3 BC cells under optimal sensing conditions. (B) DPV and (C) CV responses of the PEI@CQD@Ni-MOF-based sensor with the different concentrations of SK-BR3. Adapted/reproduced from ref. 68 with permission from Elsevier, Copyright 2025.

In a recent breakthrough, Anahita Beigi Javazm *et al.*^68^ developed a novel electrochemical biosensor for the biomarker HER2, which can detect SKBR3 breast cancer cells without the need for labels, using a hybrid nanocomposite composed of CDs, a nickel-based MOF (Ni-MOF), and polyethyleneimine (PEI). The inclusion of CDs and PEI increases the conductivity of the material, thus improving the sensitivity of the sensor. The CDs were synthesized *via* hydrothermal treatment at 130 °C using di-ammonium hydrogen citrate as a precursor. These CDs were subsequently integrated into the Ni-MOF structure through a combined process of stirring, ice-bath sonication, and heat treatment at 130 °C. Finally, the system was functionalized with PEI through prolonged incubation and dialysis, yielding the PEI@CDs@Ni-MOF nanocomposite. This hybrid material was coated onto graphite electrodes modified with reduced graphene oxide (rGO) nanoparticles, forming the active sensing interface. The anti-HER2 antibodies were immobilized on the surface, enabling the specific capture of HER2-overexpressing SKBR3 cells. Upon antigen–antibody binding, the current intensity decreased due to the restricted diffusion of the redox probe toward the electrode surface. The electrochemical behavior of this biosensor, evaluated by CV and DPV, showed a clear dependence on the concentration of SKBR3 cells (Fig. 15a–c). As the analyte concentration increased from 50 to 1000 cells per mL, the current peaks decreased due to the higher electron-transfer resistance caused by the antibody–antigen interactions on the electrode surface. A linear relationship between Δ*I* and log[*C*] was obtained (Δ*I* = 134.47 log *C* − 176.21; *R*^2^ = 0.9625), with a detection range of 100–500 cells per mL and an LOD of 1 cell per mL, confirming the excellent sensitivity and reproducibility of the PEI@CQD@Ni-MOF/rGO/GE immunosensor for HER2-positive cell detection.

From a diagnostic standpoint, the PEI@CDs@Ni-MOF/rGO/GE biosensor offers a highly efficient, low-cost strategy for the early detection of HER2-positive breast cancer. Its ability to detect individual cancer cells highlights its potential for noninvasive clinical monitoring and early-stage diagnosis. The synergistic combination of the high surface area and catalytic activity of Ni-MOFs, excellent conductivity of CDs, and biocompatibility of PEI provides a robust platform for the reliable identification of HER2-overexpressing tumor cells. This system not only simplifies fabrication and reduces costs but also achieves high reproducibility (RSD = 1.8%), long-term stability, and sustained detection capability under near-physiological conditions (pH 7.4). Its label-free design and exceptional sensitivity make it a promising tool for point-of-care testing and real-time clinical diagnostics.

Unlike the previously mentioned works, it is also possible to use optical devices for the detection of the HER2 biomarker. In this sense, in an innovative approach to the molecular diagnosis of breast cancer, Ning Li *et al.*^164^ developed a highly sensitive dual immunosensor based on a hybrid nanocomposite combining the UiO-66 MOF, CDs, and individual copper (Cu) atoms covalently anchored to defect sites in zirconium clusters within the MOF. This system was designed for the simultaneous detection of key biomarkers, which are critical for the molecular classification of breast cancer subtypes, such as HER2, ER, PR, and Ki-67. The sensing mechanism is based on a sandwich immunoassay formed by Fe_3_O_4_ magnetic nanoparticles functionalized with antibodies (Ab_1_), the target antigen, and the Cu/UiO-66 complex modified with secondary antibodies (Ab_2_). Upon antigen binding, unbound components are magnetically separated by the Fe_3_O_4_ phase, enabling a signal directly proportional to the biomarker concentration. The Cu/UiO-66 framework plays a dual role: it serves as a structural and catalytic component, exhibiting peroxidase-like activity that catalyzes the oxidation of *o*-phenylenediamine (OPD) into 2,3-diaminophenazine (DAP) in the presence of H_2_O_2_. The resulting yellow product displays an absorption peak at 425 nm, allowing colorimetric quantification of the analyte. In parallel, the system enables fluorescence detection through a ratiometric mechanism. The DAP product emits at 556 nm, selectively attenuating the blue fluorescence of the CDs at 424 nm *via* IFE. The ratio of the two emission intensities (*I*_556_/*I*_424_) provides a highly sensitive and quantifiable signal. Owing to the high fluorescence efficiency and compatibility of the CDs, this dual-mode system achieves ultrasensitive detection limits down to sub-pg mL^−1^ for all four biomarkers within the linear range of 1–1000 pg mL^−1^.

From a diagnostic standpoint, this optical platform offers a noninvasive, highly accurate approach to breast cancer subtype classification. By simultaneously quantifying HER2, ER, PR, and Ki-67 in human serum, the sensor achieved a classification accuracy of 87.5%, comparable to conventional immunohistochemical assays. This system effectively distinguishes between Luminal A, Luminal B (HER2+), and triple-negative breast cancer types, demonstrating its ability to reflect tumor heterogeneity through serum biomarker profiles. The combination of Cu single-atom catalytic sites, fluorescent CDs, and the biocompatible UiO-66 matrix results in a stable, selective, and sensitive biosensing platform suitable for real-time *in vitro* diagnosis. Its dual colorimetric-fluorescent response provides a built-in confirmation mechanism that enhances its reliability, making it a powerful tool for early breast cancer detection and molecular typing *via* liquid biopsy.

#### 4.2.3. Cancer therapy

In recent years, MOFs have emerged as versatile platforms for therapeutic applications. Their high porosity, loading capacity, structural stability, and potential for post-synthesis functionalization make them ideal for the encapsulation and controlled release of drugs or photosensitizers.^16^ Further, owing to their excellent biocompatibility and unique optical properties, CDs have emerged as promising materials in bioimaging, biosensing, and light-activated therapies.^165^ The integration of MOFs and CDs into hybrid nanocomposites allows the combination of the structural advantages of MOFs with the photoactive capabilities of the CDs, generating multifunctional systems for cancer treatment.

There are some reports on CDs@MOFs-based composites being used in therapies such as PDT, photothermal therapy (PTT), chemodynamic therapy (CDT), GSH depletion, and ICB-based immunotherapy.^74,75^ Furthermore, more advanced DDSs have been developed to optimize the bioavailability, stability, and targeted release of therapeutic agents such as DOX and ADG, whose pharmacological properties are limited by their low solubility and systemic toxicity.^166,167^ In this way, CDs@MOFs nanocomposites are emerging as promising platforms for personalized, highly effective, and minimally invasive cancer therapies.

**Fig. 16:**
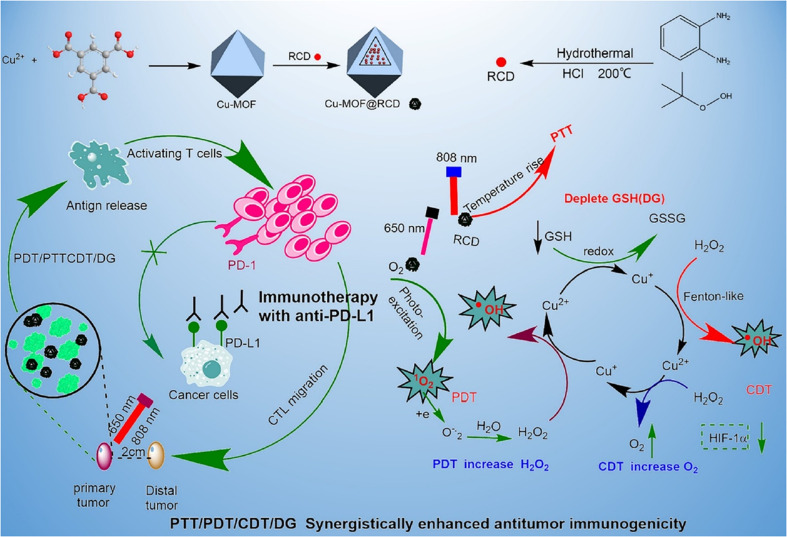
Synthesis of Cu-MOF@r-CD and its mechanism of action for PTT/PDT/CDT/DG synergistic tumor immunotherapy. Adapted/reproduced from ref. 74 with permission from the Royal Society of Chemistry, Copyright 2023.

To develop more effective cancer therapies, Zhongping Su *et al.* designed an innovative therapeutic nanocomposite called r-CDs@Cu-MOF, based on a copper MOF (Cu-MOF) combined with red-emitting carbon dots (r-CDs).^74^ These components were previously synthesized separately and then assembled by physical mixing in ethanol under stirring (Fig. 14). This material was used as a platform for multimodal ICB therapy to eradicate primary tumors, inhibit metastatic lesions, and enhance the host immune response. In this sense, Cu-MOF nanoparticles not only serve as vehicles for the controlled release of therapeutic agents but also act directly as catalysts in Fenton-type reactions, decomposing endogenous H_2_O_2_ in the TME to generate hydroxyl radicals (˙OH) and molecular oxygen (O_2_), essential for PDT and CDT. Moreover, irradiation at 650 nm activates r-CDs to generate singlet oxygen (^1^O_2_), which triggers oxidative processes and promotes the continuous formation of H_2_O_2_ and other ROS. Simultaneously, light at 808 nm induces a photothermal effect that improves tumor circulation, reduces hypoxia, and enhances the efficacy of PDT and CDT therapies. In this way, the r-CDs@Cu-MOF composite in combination with anti-PD-L1 antibodies enhances the therapeutic effect, achieving a synergistic action (PDT, PTT, CDT, DG, and ICB) effective against primary tumors, metastases, and distant lesions. Its multimodal therapeutic mechanism is shown in Fig. 16.

Another example of mitochondria-targeted PDT is the work by Qin Xiang *et al.*,^75^ who developed a highly efficient hybrid nanocomposite based on the integration of upconverting CDs and a porous PCN-224 MOF. The synthesis was carried out through a simple physical process involving mixing by sonication and stirring at room temperature for 24 hours, maintaining a 1 : 1 mass ratio between the MOF and the CDs.

**Fig. 17:**
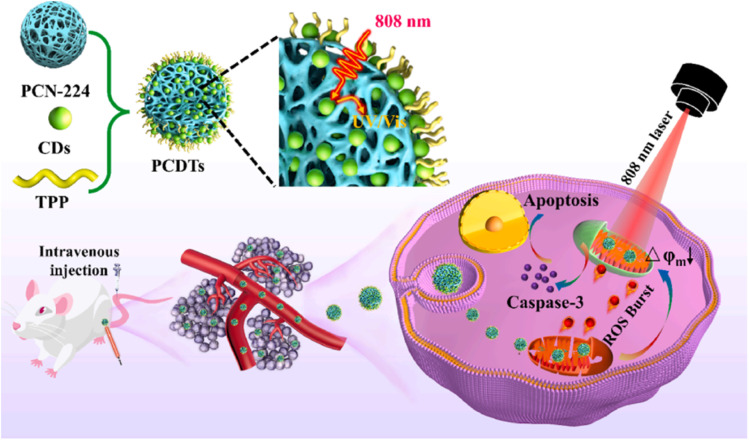
Schematic of the structure of PCDTs and their application in an 808 nm NIR light-activated and mitochondria-targeted PDT. Adapted/reproduced from ref. 75 with the permission from Elsevier, Copyright 2022.

As illustrated in Fig. 17, a nanocomposite named PCDTs was developed by integrating CDs into a porphyrin-based MOF and subsequently functionalizing its surface with triphenylphosphine (TPP) ligands to achieve mitochondrial targeting. The CDs, synthesized *via* a simple one-step hydrothermal method, displayed near-infrared PL, enabling efficient emission under 808 nm irradiation. Their ultrasmall size allowed uniform distribution both within the channels of the MOF and across its surface. Moreover, the oxygen-containing groups on the CDs established coordinative interactions with the unsaturated metal sites of the MOF, conferring structural stability and facilitating highly efficient energy transfer to the porphyrins. This synergy boosted the generation of ROS, including ^1^O_2_, essential for PDT. Through TPP functionalization, the system preferentially accumulated in the mitochondria, where localized ROS production triggered rapid mitochondrial dysfunction, intensifying the therapeutic outcome. Both *in vitro* and *in vivo* studies demonstrated potent cancer cell eradication and tumor suppression, highlighting the potential of this platform for clinical translation.

In the field of anticancer drug delivery, Hamed Alijani *et al.*^166^ developed a multifunctional nanocarrier with therapeutic and bioimaging capabilities. The platform was built on a Fe_3_O_4_@MOF core–shell structure, synthesized by growing UiO-66-NH_2_ on Fe_3_O_4_ magnetic nanoparticles in DMF at elevated temperatures. Leveraging the high porosity and loading capacity of MOFs, DOX was incorporated as the chemotherapeutic agent. The surface was then functionalized with EDC/NHS-activated CDs, endowing the system with fluorescence capability for cellular tracking. Finally, the AS1411 aptamer, which specifically binds nucleolin, was conjugated to the nanocarrier to achieve selective targeting of tumor cells. The incorporation of CDs not only provided fluorescence capability to the system but also allowed for *in vitro* evaluation of its biodistribution. At the same time, the aptamer facilitated the selective recognition of triple-negative breast cancer cells (MDA-MB-231). In this way, the Fe_3_O_4_@MOF-DOX-CDs-Apt system demonstrated efficient DOX release, the ability to induce apoptosis in tumor cells, and potential applications in cell imaging studies. Furthermore, its cytotoxicity in normal epithelial cells (HUVACs) was analyzed, supporting its viability as a promising platform for oncology therapies.

A notable example is the recent work by Wenndy Pantoja-Romero *et al.*,^167^ who designed a hybrid nanocomposite for controlled drug release in cancer therapy by combining ADG with the MOFs MIL-53(Al) and ZIF-8, along with CDs, both undoped (CBQDs) and doped (D-CBQDs) with nitrogen and sulfur. The synthesis employed a solvent-free high-pressure compression method, offering an environmentally friendly, time-efficient, and energy-saving alternative. In this process, equal proportions of ADG, MOFs, and CBQDs/D-CBQDs were mixed and subjected to 0.3 GPa for 15 minutes at room temperature using a hydraulic press.

The conjugation of ADG with CDs induced significant optical and structural changes in the nanomaterials, demonstrating an increased affinity of the drug for aqueous media. This interaction lowered the partition coefficient (log *P*) of ADG, indicating enhanced water solubility due to the incorporation of CBQDs. The high solubility of CBQDs and D-CBQDs owing to the polar functional groups on their surfaces promoted the formation of water-dispersible nanocomposites through π–π interactions. Such a property is particularly advantageous in biomedical applications, enabling improved dispersion of hydrophobic drugs, such as ADG, in aqueous environments. Unlike systems where the drug is solely encapsulated within MOFs, the inclusion of CBQDs facilitated both external anchoring and partial entrapment of ADG within the MOF pores, as confirmed by spectroscopic analyses. Preliminary cytotoxicity tests against human prostate cancer PC3 cells supported the potential of this hybrid material as a more efficient and sustainable drug delivery platform.

In summary, the combination of MOFs and CDs has demonstrated enormous potential for developing more effective and multifunctional cancer therapies, integrating controlled drug release, diagnostics, and light-activated treatments into a single platform. However, to translate these advances into the clinical setting, it is essential to further study their biocompatibility, mechanisms of action, and stability in biological media. Continued research in this field will optimize their therapeutic performance and pave the way for more personalized and sustainable cancer treatment.

### 4.3. Antibacterial activities of the nanohybrids

Bacterial infections remain a primary global health concern, significantly contributing to morbidity and mortality rates worldwide. While the introduction of antibiotics marked a turning point in combating these illnesses, their extensive and prolonged use has accelerated the emergence of antimicrobial resistance (AMR), which the World Health Organization identifies as one of the most critical health threats worldwide.^168^ Under these circumstances, the development of innovative therapeutic strategies capable of overcoming the shortcomings of traditional antibiotics has become imperative. In recent decades, research efforts have increasingly focused on alternative antimicrobial agents, particularly nanosystems with bactericidal activity. A wide range of nanomaterials, including metal nanoparticles, metal oxides, and polymers, have demonstrated broad-spectrum antibacterial activity against multiple pathogens.^169,170^ However, concerns related to their cytotoxicity and environmental persistence still limit their widespread application, prompting the development of biocompatible antibacterial systems with controlled release and reduced ecological impact. These emerging strategies aim to enhance therapeutic efficacy while contributing to sustainable approaches for mitigating AMR.

In recent years, MOFs have gained increasing interest as effective antimicrobial platforms. Owing to their crystalline and porous structures, they not only exhibit favorable biocompatibility but also allow the controlled release of active components.^171^ Their capacity to host antibacterial ions such as Ag, Cu, or Zn, along with functional organic ligands, offers distinct benefits over traditional inorganic or organic nanomaterials, which are often limited by toxicity concerns or uncontrolled ion release.^172^ Additionally, some MOFs possess intrinsic peroxidase-like activity, enabling the catalytic conversion of H_2_O_2_ into highly bactericidal hydroxyl radicals (˙OH), thereby amplifying their antimicrobial effectiveness.^173^

At the same time, although the use of CDs as antibacterial agents has been less explored, recent studies have shown that their charged surface favors electrostatic interactions with bacterial membranes, causing structural damage and cellular destabilization.^174^ Furthermore, CDs can induce the formation of ROS, disrupt the bacterial electron transport chain, and interfere with cell wall synthesis.^175^ Their ability to generate reactive radicals from their surface functional groups has also been highlighted as a key antimicrobial pathway while maintaining optical properties useful for biosensing.^176^

In addition, multifunctional CDs can inhibit microbial growth through radical generation from surface functional groups while exhibiting notable optical and electrochemical sensing properties.^177^

The integration of MOFs with CDs has opened a promising avenue for the design of advanced antibacterial nanosystems. The combination of the porous and tunable structures of MOFs with the fluorescent, photostable, and biocompatible properties of CDs allows the development of multifunctional platforms for simultaneously monitoring and eliminating bacteria.

This section reviews the studies reported to date on the use of CDs@MOFs hybrid materials as antibacterial agents. The various methodologies employed are described, highlighting the mechanisms of action in each case, including the generation of ROS and the controlled release of active agents.

#### 4.3.1. Antibacterial mechanisms and reported systems

One of the pioneering studies on the use of CDs@MOFs composites as antibacterial agents was reported by Nikolina A. Travlou *et al.*^64^ In this work, the authors designed a hybrid material by integrating an Ag-1,3,5-benzenetricarboxylate MOF (AgMOF) with sulfur- and nitrogen-doped CDs (S-CDs and N-CDs, respectively). S-CDs and N-CDs were synthesized using polystyrenesulfonate and polyvinylpyrrolidone as precursors, respectively. AgMOF was prepared from benzene tricarboxylic acid (BTC) and Ag(NO_3_) by mechanical stirring at room temperature. To obtain the hybrid materials, CDs were incorporated either during AgMOF synthesis or by introducing them into the pre-formed MOF under stirring, following a bottle-around-ship approach. Its antibacterial performance was evaluated against representative Gram-positive (*Bacillus subtilis*) and Gram-negative (*Escherichia coli*) strains. Results obtained from disk diffusion assays and minimum inhibitory concentration (MIC) tests demonstrated an apparent synergistic effect, where the hybrid system exhibited superior antibacterial activity compared to the individual components, either the MOF or the CDs alone. This improvement was partly attributed to the charge transfer from the MOF to the CDs, a phenomenon that intensified the electrostatic interaction with the bacterial membranes. In these hybrids, surface silver from the MOF is released and interacts with sulfur-containing compounds, producing Ag° and Ag_2_S species responsible for microbial inhibition. The composites contained smaller Ag nanoparticles than the pure AgMOF, enhancing their antibacterial activity, while the unmodified MOF formed larger rod-like structures that degraded slowly and released silver ions less efficiently. During composite formation, CD functional groups (sulfonates, sulfates, amines, amides, and carboxyls) coordinated with the Ag^+^ centers of the MOF. This coordination facilitated electron transfer, imparting a positive charge to the framework, which enhanced its interactions with bacterial membranes and reinforced its inhibitory effect. The resulting antimicrobial activity was comparable to or even greater than that of certain antibiotics while preserving good cell viability at low concentrations.

As noted above, one of the antibacterial mechanisms of MOFs involves the release of active agents, either through the liberation of the metal centers or the organic ligands, thereby exerting the antimicrobial effect. In this context, Jing He *et al.*^76^ developed a hybrid material with antimicrobial properties from a three-dimensional zeolitic imidazolate framework nanosheet (ZIF-L) with a flower-like morphology and N-CDs, obtaining the N-CDs@ZIF-L composite. ZIF-L was synthesized from Zn(NO_3_)_2_ and Hmim in an aqueous solution by stirring. N-CDs were produced *via* a one-step hydrothermal method using citric acid and diethylenetriamine (DETA). Finally, the N-CDs@ZIF-L composites were prepared by physically mixing ZIF-L and N-CDs in water under stirring at room temperature. ZIF-L shares the same basic structural unit as ZIF-8 but has a different three-dimensional extension. Both ZIF-L and ZIF-8 exhibit notable antibacterial activity through the release of Zn^2+^ ions and imidazole-based ligands, which contribute to microbial inhibition. However, ZIF-L displays superior antimicrobial performance compared to ZIF-8, as its unique laminar structure with sharp edges can physically disrupt bacterial membranes. The results indicated that the N-CDs@ZIF-L composite is more stable in aqueous media and exhibits better antimicrobial performance than pure ZIF-L. Tests against *E. coli* and *S. aureus* showed efficacies exceeding 95% and 85%, respectively, with an MIC of 0.125 mg mL^−1^ after incorporation of N-CDs. This improvement was associated with increased surface roughness and greater stability, albeit with a slight decrease in the surface charge. To explore practical applications, they were incorporated into polylactic acid (PLA), a biocompatible and biodegradable polymer widely used in medicine, by solution casting, obtaining films with clear antibacterial properties.

The use of CDs@ZIF-8-based composites as an antibacterial agent was also reported by Jahanghir Azizi *et al.*^69^ In this case, the CDs were obtained from marshmallow leaf extract through a process that included hydroalcoholic extraction, sonication, and a hydrothermal reaction. ZIF-8 was subsequently formed in the presence of the CDs using 2-MeIM and Zn(NO_3_)_2_ as precursors.

**Fig. 18:**
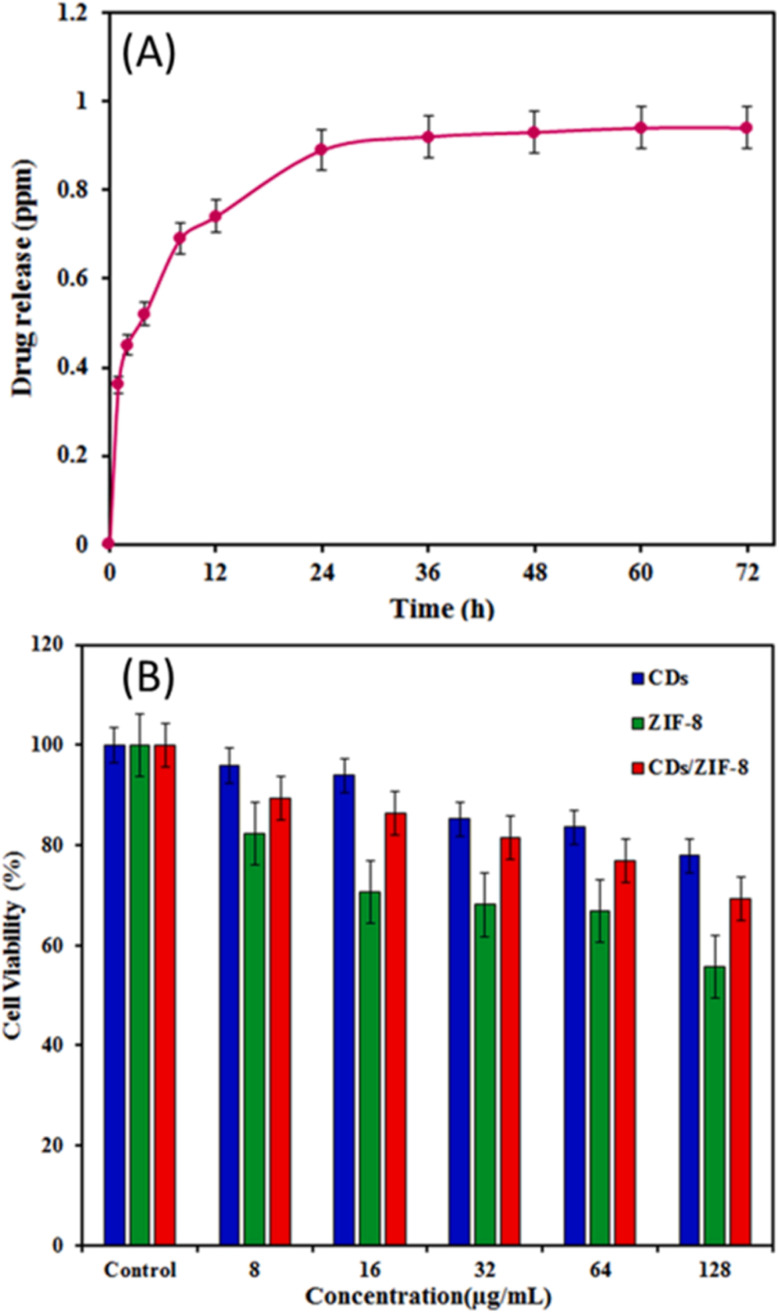
(A) *In vitro* 2-MeIM release from the CDs/ZIF-8 nanocomposite at pH 7.4. (B) Cell viability of HFF-2 cells after 48 treatments with different concentrations of ZIF-8, CDs, and the CDs/ZIF-8 nanocomposite. Adapted/reproduced from ref. 69 with permission from Elsevier Science, Copyright 2025.

The release behavior of the encapsulated 2-MeIM from the CDs@ZIF-8 nanocomposite (Fig. 18a) revealed a rapid initial stage followed by a sustained-release profile, reaching equilibrium after approximately 48 h. This pattern confirms the controlled-release capacity of the hybrid system, which is crucial for maintaining prolonged antimicrobial activity. The gradual diffusion of 2-MeIM, together with the potential release of Zn^2+^ ions, provides continuous antibacterial action over time. In addition, cytotoxicity testing on HFF-2 human dermal fibroblast cells (Fig. 18b) showed high cell viability (>75%) even at concentrations up to 128 µg mL^−1^, confirming the biocompatibility of the nanocomposite. The CDs@ZIF-8 hybrid exhibited lower cytotoxicity compared to pristine ZIF-8 owing to the passivating effect of the CDs, which reduced Zn^2+^-induced toxicity and improved surface compatibility with biological tissues.

Another mechanism of antibacterial activity corresponds to the production of ROS, which can induce oxidative damage and compromise the integrity of cell membranes, leading to bacterial death. In this context, Ayush Amod *et al.*^178^ reported the development of a hybrid nanomaterial, termed nanocluster–carbon dots (NCCDs), composed of CDs and fluorescent copper nanoclusters (CuNCs), obtained using a MOF as a structural scaffold. The CuNCs were synthesized within a MOF matrix derived from ZnSO_4_ and terephthalic acid, followed by the incorporation of CuSO_4_ and subsequent UV irradiation. At the same time, CDs were obtained through microwave-assisted heating of a mixture of diethylene glycol (DEG) and polyethyleneimine (PEI). In this case, CDs and CuNCs act through multiple mechanisms, including mechanical and physical disruption of the bacterial membrane and the generation of ROS. As a result, NCCDs exhibited broad-spectrum antibacterial activity, with MIC_90_ values of 20 ± 2, 7.5 ± 2, and 10 ± 3 µg mL^−1^ against *Escherichia coli*, *Pseudomonas aeruginosa*, and *Bacillus subtilis*, respectively. The nanocomposite inhibited biofilm formation in *B. subtilis* and *P. aeruginosa* by 40.27% and 68.10%, respectively, and reduced the biomass of established biofilms by 53.09% and 68.43%, respectively. Antibiofilm efficacy was further enhanced when combined with matrix-degrading enzymes because such a mechanism involves membrane perforation, leading to osmotic imbalance. Moreover, NCCDs were found to promote the transition of bacteria from biofilm to planktonic states, thereby increasing their susceptibility to conventional antibiotics. In addition, the hybrid material demonstrated the ability to eliminate biofilms formed on the surfaces of medical implants. These results position NCCD as a promising candidate for combating resistant and biofilm-associated bacterial infections.

**Fig. 19:**
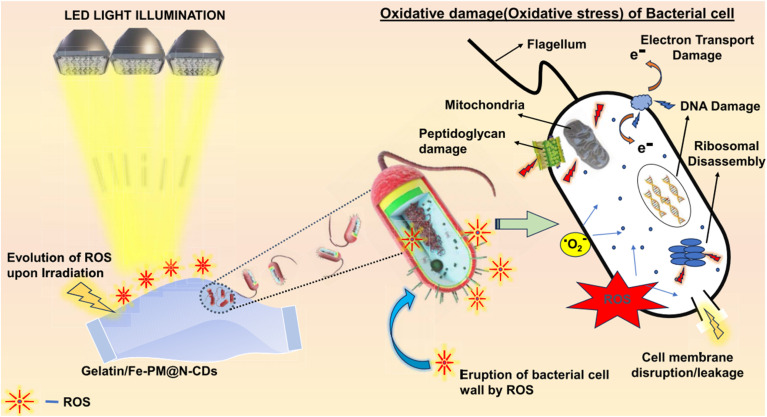
Antibacterial mechanism of the composite film. Adapted/reproduced from ref. 77 with permission from the American Chemical Society, Copyright 2025.

Another strategy for generating ROS is photodynamic inactivation (PDI), which has emerged as an innovative and highly effective approach for microbial control. Building on this concept, P. Ananthi *et al.*^77^ developed a hybrid material consisting of an iron-based porphyrin MOF (Fe-PM), nitrogen-doped carbon dots (N-CDs), and gelatin, designed to exhibit strong antibacterial activity against both Gram-negative (*Escherichia coli*) and Gram-positive (*Staphylococcus aureus*) bacteria. The N-CDs were synthesized from silk cocoons *via* a hydrothermal method, while the Fe-PM was obtained through a solvothermal process using FeCl_3_·6H_2_O and TCPP in a mixed solvent of DMF and ethanol. Finally, the Fe-PM@N-CD composite was assembled by ultrasonic treatment. As shown in Fig. 19, the antimicrobial mechanism is based on the photodynamic effect, where exposure to LED light activates the photosensitizing centers of the Fe-PM@N-CDs composite, triggering the generation of ROS such as singlet oxygen (^1^O_2_) and superoxide radicals (˙O_2_^−^). These ROS induce oxidative stress in bacterial cells, leading to disruption of the cell membrane, peptidoglycan degradation, and damage to essential biomolecules such as DNA and ribosomal structures. The resulting structural collapse and leakage of intracellular contents ultimately cause bacterial death.

The synergistic interaction between N-CDs and the Fe-PM framework enhances ROS generation. At the same time, incorporating gelatin improves stability and biocompatibility, making the system suitable for photoactive antibacterial and wound-healing applications.

These reactive species can irreversibly damage bacterial cells. This mechanism is nonspecific, highly effective against a wide range of pathogens, and carries a low risk of promoting bacterial resistance. Although the MOF Fe-PM stands out in PDI due to its high chemical stability, biocompatibility, and ability to produce ROS, its limited ^1^O_2_ generation restricts its performance. To overcome this limitation, a FRET system was designed in this study, in which N-CDs act as donors and Fe-PM as an acceptor. The incorporation of N-CDs significantly enhanced the generation of ^1^O_2_, as confirmed in specific tests, notably increasing the photodynamic activity of the compound. The gelatin films with Fe-PM@N-CD (GPMCD) displayed optimized properties, including increased mechanical strength, 68% biodegradability, and outstanding antibacterial activity, achieving 97% inactivation of bacteria in just 10 min under white LED illumination. Although this novel material is intended for packaging fresh green grapes, its antibacterial properties open the door to potential biomedical applications. The antibacterial activity of CDs@MOFs hybrid materials highlights their potential as promising candidates for wound dressings, effectively inhibiting pathogenic bacteria while simultaneously fostering a favorable environment for tissue repair.

#### 4.3.2. Wound-healing and biomedical applications

Beyond their broad-spectrum antibacterial activity, several CDs@MOFs-based composites have exhibited remarkable promise in biomedical applications, particularly in the treatment of infected and chronic wounds. Such wounds remain a major healthcare challenge due to bacterial colonization, delayed tissue regeneration, and persistent inflammation. In this scenario, multifunctional antimicrobial materials that simultaneously prevent infection and promote tissue repair have attracted increasing attention.

CDs@MOFs hybrid systems combine the advantages of MOFs and CDs, enabling synergistic effects such as catalytic ROS generation, controlled drug release, and biocompatible photothermal or photodynamic responses.^70,179–182^ These properties make them excellent candidates for accelerating healing while minimizing infection and inflammation.

In this context, Swarup Krishna Bhattacharyya *et al.*^179^ developed an innovative system based on vitamin B12-loaded gelatin microspheres coated with a CD-functionalized MOF (CDs@FMOF) as a wound dressing material with antibacterial activity. The CDs were obtained by microwave-assisted pyrolysis from citric acid, while the FMOF was synthesized in dimethylformamide using Zn(CH_3_COOH)_2_, 2-MeIm, and 2-aminobenzimidazole (2-ABIM) in DMF, incorporating the CDs by physical mixing. Vitamin B12 was subsequently loaded into the FMOF by physical stirring, and the resulting material was coated with gelatin microspheres formed by a water-in-oil emulsion and crosslinked with 1-(3-dimethylaminopropyl)-3-ethyl carbodiimide and *N*-hydroxysuccinimide (EDC/NHS), giving rise to [(FMOF/B12)@MS]. The final structure showed a high specific surface area, hierarchical porosity, and an abundance of carboxyl and amino groups sensitive to pH variations, which also allowed local environmental monitoring capabilities. The synergistic combination of Zn^2+^ ions, imidazole units, and CDs generated an outstanding bactericidal effect against *Escherichia coli* and *Staphylococcus aureus*. These components favored the destabilization of bacterial membranes and enhanced the antimicrobial action, positioning the material as a promising alternative against wound infections. In parallel, the gelatin coating enabled a controlled release profile of vitamin B12, which stimulated the proliferation of L929 fibroblasts and promoted tissue regeneration in *in vitro* models.

**Fig. 20:**
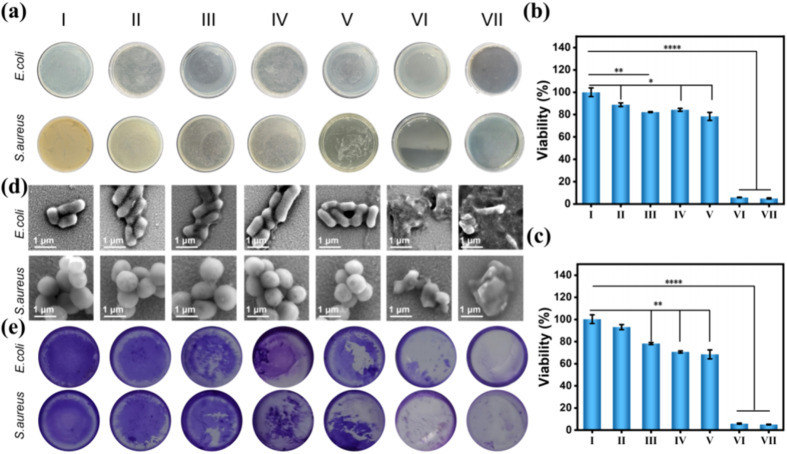
(a) In the disk diffusion assay, the antibacterial plates of each experimental group were used against *E. coli* and *S. aureus*. Quantitative graph of antibacterial activity against (b) *E. coli* and (c) *S. aureus* for each experimental group in the disk diffusion assay. (d) SEM images of the antibacterial effects of each experimental group on *E. coli* and *S. aureus* in the disk diffusion assay. (e) Crystal violet-stained biofilm images of samples treated with various regimens (experimental groups: I: PBS; II: L-Arg; III: Arg-CDs; IV: Arg-CDs/ZIF-8; V: H_2_O_2_; VI: Arg-CDs + H_2_O_2_; VII: Arg-CDs/ZIF-8 + H_2_O_2_). Adapted/reproduced from ref. 180 with permission from the American Chemical Society, Copyright 2025.

Another noteworthy study that was related to ZIF-8 was conducted by Qi Tao *et al.*,^180^ who designed a sustained-release antibacterial nanoenzyme system by incorporating arginine-doped carbon dots (Arg-CDs) into a ZIF-8 framework as a non-antibiotic therapeutic platform for the treatment of wound infections. ZIF-8 was synthesized through the reaction of Zn(NO_3_)_2_ and 2-MeIm in methanol, while the Arg-CDs were prepared *via* a hydrothermal process using l-arginine and ethylenediamine. Both components were then integrated through physical mixing in methanol, yielding the Arg-CDs@ZIF-8 nanocomposite. As shown in Fig. 20, this hybrid system exhibited strong antibacterial activity against both *E. coli* and *S. aureus*. The disk diffusion assay (Fig. 20a) and viability analyses (Fig. 20b and c) demonstrated that the Arg-CDs@ZIF-8 composite in the presence of H_2_O_2_ achieved nearly complete bacterial inhibition (≈95%), significantly surpassing the performance of its individual components. SEM micrographs (Fig. 20d) revealed severe morphological damage in bacterial membranes after treatment, while crystal violet staining (Fig. 20e) confirmed effective biofilm disruption. The antibacterial mechanism involves the pH-responsive release of Arg-CDs from the ZIF-8 framework, which, in acidic environments, catalyzes the decomposition of H_2_O_2_ to generate ROS and nitric oxide (NO). These species synergistically damage bacterial membranes and internal components, leading to rapid cell death. *In vivo* tests further evidenced accelerated epidermal regeneration and wound closure, confirming the potential of the composite as a biocompatible and efficient therapeutic agent for infected wound healing.

In the search for new materials with antimicrobial properties, PDT has attracted significant interest as it allows the generation of ROS. In this context, Yang Wang *et al.*^181^ developed a hybrid aerogel patch consisting of two-dimensional (2D) sheets of a porphyrin metal–organic framework (CuTCPP-MOF) modified with CDs and incorporated into a matrix of gelatin methacrylate (GelMA) and polyacrylamide (PAM). The CDs were obtained by microwave synthesis from citric acid and ethylenediamine. Meanwhile, the CuTCPP nanosheets were prepared using solvothermal reaction using Cu(NO_3_)_2_·3H_2_O, TFA, polyvinylpyrrolidone (PVP), and tetrakis(4-carboxyphenyl)porphyrin (TCPP) in a DMF/ethanol mixture. To obtain the CuTCPP@CQDs composite, the CDs were incorporated during the synthesis process, achieving their homogeneous integration into the MOF structure. This composite was subsequently dispersed in a GelMA/PEGDA/PAM matrix and subjected to photopolymerization under UV irradiation, resulting in the CDs@ GPP/CuTCPP patches. CuTCPP-type porphyrin MOFs possess high surface area and light absorption capacity but show limited ROS generation. The incorporation of CDs, acting as biocompatible energy donors with high quantum yield, enhances FRET processes and markedly improves photodynamic performance. The hybrid material demonstrated potent photodynamic antibacterial action, with kill rates exceeding 99.99% against *Staphylococcus aureus* and *Escherichia coli* within 1 hour of irradiation. In addition to its antimicrobial capacity, the aerogel demonstrated high porosity and good fluid absorption, allowing it to form a physical barrier over the wound, promoting coagulation and preventing secondary infections.

Materials with antibacterial properties applied in wound healing have also proven highly effective in the treatment of diabetic wounds. These chronic lesions represent one of the most severe complications of the disease, as their healing is impaired by a hyperglycemic microenvironment that fosters bacterial proliferation and delays tissue repair. In this way, Shuxin Zhang *et al.*^70^ developed a multifunctional wound dressing based on glucose oxidase-based nanofibers/CDs anchored to a copper MOF (GOx/CDs@MOF NFs) with antibacterial action, designed to combat infections and promote diabetic wound healing. The CDs were obtained *via* hydrothermal synthesis using ciprofloxacin hydrochloride as a precursor. The CDs@MOF hybrid was synthesized *via* a co-assembly approach, in which a CDs solution was incorporated during the solvothermal synthesis of the MOF using Cu(NO_3_)_2_·3H_2_O and H_3_BTC as precursors. Glucose oxidase (GOx) was then immobilized onto the composite through physical stirring. The resulting system was embedded in a PVB matrix by electrospinning, yielding porous nanofibers with high biocompatibility and a three-dimensional architecture resembling the extracellular matrix. The authors point out that the mechanism of action of this material is based on the ability of GOx to catalyze the conversion of endogenous glucose into gluconic acid and H_2_O_2_. The gluconic acid-induced pH decrease enhances the peroxidase-like activity of the Cu-MOF, promoting the transformation of H_2_O_2_ into highly bactericidal hydroxyl radicals (˙OH). This catalytic process showed remarkable antibacterial efficacy in both *in vitro* and *in vivo* models, where the nanofibers not only inhibited microbial growth but also accelerated tissue regeneration. Furthermore, the CDs integrated into the nanofibers conferred pH-dependent fluorescent responsiveness, with stable and reversible signals in the range of 5.0–9.5. These fluorescence variations can be captured with a smartphone and analyzed using RGB values, enabling direct, real-time monitoring of the wound status.

In the treatment of diabetic ulcers, one of the main challenges is the coexistence of chronic inflammation, excess ROS, and recurrent bacterial infections, all of which delay healing and complicate recovery. To address these limitations, Sheng Dai *et al.*^182^ developed an innovative hybrid material based on the ZIF-8 metal–organic framework and copper-doped carbon dots (Cu/CDs). The Cu/CDs were obtained using citric acid, guanidine, and CuCl_2_ through a hydrothermal process and subsequently integrated into a ZIF-8 MOF synthesized with 2-MeIm and Zn(CH_3_COOH)_2_ under stirring conditions. In this system, the controlled release of Zn^2+^ during ZIF-8 degradation in inflamed environments acts as a highly effective antibacterial agent, capable of disrupting the bacterial membrane and causing cell lysis. In parallel, Cu/CDs confer high catalytic activity with enzyme-mimicking functions similar to those of SOD and CAT enzymes, promoting the scavenging of free radicals and reducing oxidative damage.

The design of the CDs@ZIF-8-Cu nanocomposite prevents the rapid elimination of nanoenzymes in the inflammatory microenvironment, improves its biocompatibility, and allows for the sustained release of both Cu/CDs and Zn^2+^. This integration generates a synergistic effect that combines antioxidant action with potent antibacterial activity. *In vitro* assays demonstrated inhibition rates exceeding 90% against *Staphylococcus aureus* and *Escherichia coli*, along with a significant reduction in biofilm formation.

*In vivo* studies supported these results, showing that the nanocomposite not only reduces inflammation at the wound site but also stimulates vascular regeneration, promotes collagen deposition, and accelerates healing. Together, these properties position CDs@ZIF-8-Cu as a highly promising material for the treatment of chronic wounds, offering dual functionality of combating resistant bacterial infections and controlling local oxidative stress.

In summary, CDs@MOFs composites represent a promising emerging strategy to combat resistant and bacterial infections. By combining the properties of MOFs and CDs, these hybrids offer synergistic antibacterial mechanisms such as ROS generation and sustained release of active species. Beyond their antimicrobial potential, they also show great promise in wound-healing applications, particularly for the treatment of diabetic patients. Altogether, these features position CDs@MOFs composites as an effective and safe alternative to conventional antibiotics.

## 5. Conclusions and perspectives

CDs@MOFs composites represent an emerging class of hybrid materials with tunable structural and optical properties, positioning them as up-and-coming platforms for biomedicine and biosensing. The combination of the high porosity, large surface area, and active sites of MOFs with the biocompatibility, optical stability, and versatile functionality of CDs generates unique synergistic effects.

As discussed in this review, the properties and activities of CDs@MOFs strongly depend on their synthesis method, which may vary in terms of precursors, solvents, employed strategies (one-step or two-step methods), and experimental parameters such as temperature and reaction time. Despite the notable progress, precise control over the distribution, growth, and location of CDs within the framework remains challenging, making the synthesis of CDs@MOFs a critical issue. In this context, the development of more straightforward, more sustainable, and cost-effective techniques, aligned with the principles of green chemistry, will be essential to facilitate their scalability and practical application. Likewise, diversifying the types of MOFs and carbon-based materials used, together with deeper investigation into their formation mechanisms and interfacial interactions, will enable the design of composites with functionalities tailored to specific biomedical requirements. It is also essential to define and understand the required properties according to the intended application. In this regard, this review has addressed the development and potential of these hybrid materials in biosensing, cancer diagnosis and therapy, as well as antibacterial applications.

In the field of biosensing, CDs@MOFs composites have demonstrated remarkable versatility for ultrasensitive detection of biomarkers, drugs, and other clinically relevant analytes. The combination of their tunable fluorescence with the selective adsorption capacity of MOFs has enabled the development of sensors with high sensitivity, selectivity, and reusability.

Moreover, the integration of advanced techniques such as ratiometric sensing, along with the use of different photoelectronic processes between MOFs and CDs, opens new perspectives for their application in early diagnosis and disease monitoring. Nevertheless, significant challenges remain regarding their stability in biological media, selectivity in complex matrices, and a more detailed understanding of their sensing mechanisms, which require further experimental and theoretical studies.

In the anticancer field, CDs@MOF composites provide multifunctional platforms capable of integrating controlled drug release, imaging-based diagnosis, and light-activated therapies. Their ability to modulate drug release and generate ROS under irradiation makes them ideal systems for combined targeted therapy strategies. However, clinical translation requires a deeper understanding of their mechanisms of action, stability in biological environments, and long-term safety. A key goal in this direction is to extend the emission of both CDs and MOFs into the red or near-infrared region, thereby obtaining more biocompatible materials suitable for *in vivo* applications.

Regarding antibacterial activity, CDs@MOFs exhibit complementary mechanisms that include ROS generation, cell membrane disruption, and the sustained release of metal ions. These features provide effectiveness against resistant bacteria and biofilms while also offering added value in wound healing, particularly in diabetic patients. Thus, they have emerged as an effective and safe alternative to conventional antibiotics. Nevertheless, this area remains relatively underexplored, necessitating optimization of several aspects to enhance their antibacterial performance, such as improving stability in biological environments, increasing selectivity toward specific pathogenic strains, controlling ion and active species release kinetics, and minimizing potential cytotoxic effects on healthy cells. Furthermore, molecular-level interactions with bacteria and efficacy against multidrug-resistant infections under *in vivo* conditions require further investigation.

Although some applications of CDs@MOFs demand biocompatibility and negligible toxicity, no studies have specifically focused on the toxicity of these materials. However, biological behavior of each material separately is well documented and can be used to rationalize interaction of CDs@MOFs with living organisms. Hence, the presence of metals in CDs or MOFs that can generate ROS is a common source of cellular damage, while ligand or functional group degradation may release toxic molecules. In this way, the careful design of CDs@MOFs for biomedical applications must consider including biocompatible units that form a stable structure and achieve high stability. Biological studies of CDs@MOFs, such as CDs@ZIF-8, show low cytotoxicity, highlighting how the selection of stable structures with biocompatible elements can be employed for the desired bioapplication.

Overall, CDs@MOFs constitute a hybrid platform with enormous potential in biomedicine and biosensing. Although current limitations related to biological stability, scalability, and production cost persist, research efforts aimed at understanding their synergies, optimizing synthesis, and broadening functionalities open a promising horizon for their future clinical application, spanning early diagnosis, personalized anticancer therapies, and advanced antimicrobial treatments.

## Author contributions

T. P. B.; conceptualization, investigation, methodology, validation, visualization, writing – original draft, writing – review & editing. L. L.; conceptualization, investigation, methodology, validation, visualization, writing – review & editing. D. P. S.; conceptualization, image formation, validation, visualization, writing – review & editing.

## Conflicts of interest

There are no conflicts to declare.

## Data Availability

No primary research results, software or code have been included and no new data were generated or analyzed as part of this review.
